# SPIB and BATF provide alternate determinants of IRF4 occupancy in diffuse large B-cell lymphoma linked to disease heterogeneity

**DOI:** 10.1093/nar/gku451

**Published:** 2014-05-28

**Authors:** Matthew A. Care, Mario Cocco, Jon P. Laye, Nicholas Barnes, Yuanxue Huang, Ming Wang, Sharon Barrans, Ming Du, Andrew Jack, David R. Westhead, Gina M. Doody, Reuben M. Tooze

**Affiliations:** 1 Section of Experimental Haematology, Leeds Institute of Cancer and Pathology, University of Leeds, Leeds, UK; 2 Bioinformatics Group, School of Molecular and Cellular Biology, University of Leeds, Leeds, UK; 3 Division of Molecular Histopathology, Department of Pathology, University of Cambridge, Cambridge, UK; 4 Haematological Malignancy Diagnostic Service, Leeds Cancer Centre, Leeds Teaching Hospitals NHS Trust, Leeds, UK

## Abstract

Interferon regulatory factor 4 (IRF4) is central to the transcriptional network of activated B-cell-like diffuse large B-cell lymphoma (ABC-DLBCL), an aggressive lymphoma subgroup defined by gene expression profiling. Since cofactor association modifies transcriptional regulatory input by IRF4, we assessed genome occupancy by IRF4 and endogenous cofactors in ABC-DLBCL cell lines. IRF4 partners with SPIB, PU.1 and BATF genome-wide, but SPIB provides the dominant IRF4 partner in this context. Upon SPIB knockdown IRF4 occupancy is depleted and neither PU.1 nor BATF acutely compensates. Integration with ENCODE data from lymphoblastoid cell line GM12878, demonstrates that IRF4 adopts either SPIB- or BATF-centric genome-wide distributions in related states of post-germinal centre B-cell transformation. In primary DLBCL high-*SPIB* and low-*BATF* or the reciprocal low-*SPIB* and high-*BATF* mRNA expression links to differential gene expression profiles across nine data sets, identifying distinct associations with SPIB occupancy, signatures of B-cell differentiation stage and potential pathogenetic mechanisms. In a population-based patient cohort, SPIB^high^/BATF^low^-ABC-DLBCL is enriched for mutation of MYD88, and SPIB^high^/BATF^low^-ABC-DLBCL with MYD88-L265P mutation identifies a small subgroup of patients among this otherwise aggressive disease subgroup with distinct favourable outcome. We conclude that differential expression of IRF4 cofactors SPIB and BATF identifies biologically and clinically significant heterogeneity among ABC-DLBCL.

## INTRODUCTION

Classification based on gene expression has linked clinical response to molecular biology in diffuse large B-cell lymphoma (DLBCL), an aggressive and common form of human lymphoma. The cell of origin classification has become a prevailing paradigm, and divides DLBCL into groups based on their relationship to normal B-cell counterparts: the germinal centre B-cell (GCB) and the activated B-cell type (ABC) (1). ABC-DLBCL has a worse prognosis on currently standard immunochemotherapy regimen R-CHOP (rituximab, cyclophosphamide, hydroxy-daunorubicin, Oncovin, prednisolone) and is related to the continuum of activated cell states that lie between B-cells and plasma cells. This continuum is linked to a reorganizing transcriptional network driven by changes in expression of core transcriptional regulators. We reasoned that variation in expression of these transcriptional regulators might equally contribute to heterogeneity within ABC-DLBCL.

Interferon regulatory factor 4 (IRF4) is a defining feature of ABC-DLBCL and in normal B-cells is essential for the initiation of plasma cell differentiation (2–5). The DNA-binding domain of IRF4 is restricted via an autoinhibitory interaction (6), and release depends primarily on binding to transcription factor partners. Two principle cofactors of IRF4 are the ETS-family proteins PU.1 and SPIB, at ETS/IRF Composite Elements (EICE) (7–9). While highly related, SPIB and PU.1 are only partially redundant and are essential for mature B-cell survival (10–12). SPIB can additionally act to prevent plasma cell differentiation by repressing *PRDM1*/*BLIMP1* (13). In ABC-DLBCL SPIB is of particular relevance as this gene can be subject to deregulation by amplification or translocation leading to heterogeneity in *SPIB* expression (14,15). A recent study reported on the role of SPIB in ABC-DLBCL using biotin-tagged SPIB for ChIP-seq assays, and concluded that SPIB/IRF4 heterodimers were central to ABC-DLBCL pathogenesis potentially regulating B-cell receptor signalling pathways and interferon-α (IFNα) secretion downstream of *MYD88* mutations (16). However, the contribution of endogenous partners to regulatory element usage by IRF4 was not directly assessed.

BATF, an AP1-family protein (17), was recently described as a principle IRF4 partner at AP1/IRF Composite Elements (AICEs) in T-cells and dendritic cells (18–21). This partnership was also observed in cytokine stimulated B-cells (19,20). BATF plays an essential role in both T- and B-cells during humoral immune responses, with a requirement in the germinal centre and the regulation of class-switch recombination via *AICDA* (22,23). However, in the context of B-cell malignancy BATF is consistently associated with ABC-DLBCL, representative of a post-germinal centre state, rather than GCB-DLBCL (24); BATF thus provides a potential alternate partner for IRF4 in this context.

Here, we have addressed the relationship between IRF4 and its endogenous partners in ABC-DLBCL. Our results demonstrate that SPIB does provide the functionally dominant IRF4 partner in ABC-DLBCL with SPIB deregulation, however, in this context BATF provides an alternative IRF4 partner genome-wide. We find that in primary ABC-DLBCL, variation in the expression of SPIB and BATF is associated with clinical and biological heterogeneity. Strong expression of SPIB relative to BATF is linked with better overall survival, *MYD88* mutations and expression of genes associated with SPIB occupancy and B-cell rather than plasmablast or plasma cell state.

## MATERIALS AND METHODS

### Antibodies and primers

Antibodies used were: IRF4 antibody (sc-28696X), PU.1 antibody (sc-352X), BATF antibody (sc-100974X, Santa Cruz), BLIMP1 polyconal antibody as described (25), monoclonal anti-β-ACTIN (clone AC-15, Sigma), rabbit anti-mouse immunoglobulin G (IgG; Jackson ImmunoResearch), control rabbit IgG (Upstate Biotechnology), control mouse IgG (Sigma), anti-MYC clone 9E10.

### Vectors and antibody generation

Coding sequence for human SPIB a.a.1–51 was cloned into pGEX6P1 between EcoRI and BglII and sequence verified, for primers see Supplementary Methods. GST-fusion proteins were expressed according to manufacturer's instructions (Amersham) and used to generate rabbit polyclonal antisera according to standard procedures (Harlan Seralab).

Myc-epitope tagged coding sequence for human SPIB, SPI1(PU.1) and IRF4 were cloned into pIRES2EGFP (Clontech) between EcoRI and BglII for SPIB, EcoRI and BamHI for SPI1/PU.1 and IRF4, and sequence verified.

### Cell lines, culture, transfection and knockdown

H929, HeLa and COS cells were cultured in RPMI1640 media and OCI-LY3, OCI-LY10 (kind gift of Prof. R.E. Davis) in Iscove's Modified Dulbecco's Medium (IMDM) with GlutaMAX™ (Life Technologies™), each containing 10% heat inactivated fetal calf serum. COS and HeLa cells were transfected with GeneJuice (Novagen) according to the manufacturer's instructions.

### Western blot, ChIP and Electrophoretic Mobility Shift Assay

Western blots were performed according to standard procedures. ChIP and electrophoretic mobility shift assay (EMSA) were performed as described (26). For BATF the ChIP method was adapted such that protein A Sepharose (Thermo Scientific) was first saturated with rabbit anti-mouse secondary antibody, and then incubated with anti-BATF or control mouse IgG. Pre-bound beads were used to immunoprecipiate chromatin fractions. Nuclear extracts for EMSA were prepared from transfected COS cells, and OCI-LY3 and -LY10 cell lines. For EMSA probe sequences and ChIP PCR primer sequences see Supplementary Methods.

### Library preparation and sequencing

Library preparation for input chromatin, IRF4, SPIB and PU.1 was performed using the Illumina ChIP-seq Sample Prep Kit (Illumina^®^) according to manufacturer's instructions, and run on a GAIIx Genome Analyser (Illumina^®^, Little Chesterford, UK). BATF samples, and libraries generated for control siRNA and SPIB siRNA treated chromatin were prepared using the MicroPlex Library Preparation™ kit (Diagenode) for ChIP, size selected using AMPure XP beads (Beckman Coulter) and run on an Illumina Hiseq 2500.

### siRNA knockdown, RNA extraction and gene expression analysis

OCI-LY3 and OCI-LY10 cells were transfected with SPIB siRNA (s13354; 4392420—Ambion Life Technologies™) or control non-targeting siRNA, (27) using Amaxa^®^ Nucleofector^®^ system and Amaxa^®^ Cell line Nucleofector^®^ Kit V (Lonza), setting D.023, according to manufacturer's instructions. RNA was extracted with TRIzol^®^ and amplified using Illumina^®^ TotalPrep™-96 RNA Amplification Kit (Life Technologies™). Resulting cRNAs were then hybridized to BeadChips using the HumanHT-12 v4 Expression BeadChip Kit according to manufacturer's instructions, and the BeadChips scanned with the Illumina BeadArray Reader (Illumina^®^). Analysis was performed as previously described (28).

### MYD88 mutation screening

Primary DLBCL samples related to GSE32918 were retrieved from the Haematological Malignancy Diagnostic Service of Leeds Teaching Hospital NHS Trust. DNA from representative sections was extracted using standard proteinase K digestion and the QIAamp DNA Micro Kit (QIAGEN, Crawley, UK). *MYD88* gene mutation was screened by PCR and Sanger sequencing. The primer sequences and PCR conditions are detailed in Supplementary Methods. PCR products were sequenced using the BigDye Terminator v3.1 (Applied Biosystems, Foster City, CA, USA). Sequence changes were confirmed by at least two independent PCR and sequencing experiments. The somatic mutation was ascertained by excluding germline changes through database search and analysis of DNA from microdissected normal cells.

### Data sets and analysis

A set of 10 DLBCL data sets were used as previously described (24), including data sets derived from the Gene Expression Omnibus: GSE32918, GSE10846, GSE12195, GSE19246, GSE22470, GSE22895, GSE31312, GSE34171, GSE4475, (29–37) as well as the data of Monti *et al.* (10), and Wright *et al.* (http://llmpp.nih.gov/DLBCLpredictor/) (38). The data set, GSE10846, was split into treatment groups (CHOP/R-CHOP) yielding two data sets that were then analysed independently (referred to as GSE10846_CHOP and GSE10846_R-CHOP). Data set GSE41208 covering progressive changes in gene expression during plasma cell differentiation was previously described (28). Data generated for this manuscript are available via GSE50015 and GSE56857.

### ChIP-seq data analysis and motif detection

For more detail see Supplementary Methods. Trimmed reads were aligned with Bowtie2 (39), and analysed for peaks using GEM (40). Peak overlaps were determined using a clustering approach such that any peak centre <250 bp from an index peak centre were considered part of an overlapping cluster. *De novo* motif detection was performed with HOMER (41). Displayed motifs are provided as matrices in Supplementary Table S2. The Broad IGV tool was used to display ChIP-seq data (42,43).

## RESULTS

### SPIB, PU.1 and IRF4 cis-regulatory occupancy in ABC-DLBCL cell lines

Assessment of endogenous SPIB is essential in order to understand how distinct cooperating factors contribute to IRF4 regulatory element usage in ABC-DLBCL. We therefore raised a polyclonal antibody against the variable amino-terminus of the protein, which did not cross-react with PU.1 or SPIC, and was validated in conventional ChIP assays (Supplementary Figure S1A–C). We then performed ChIP-seq for SPIB, PU.1 and IRF4 from the ABC-DLBCL cell lines OCI-LY3 and OCI-LY10. We identified 6379 and 13184 IRF4 sites, 2937 and 8904 PU.1 sites and 21055 and 14234 SPIB sites in OCI-LY3 and LY10, respectively (Supplementary Table S1).

**Figure 1.**
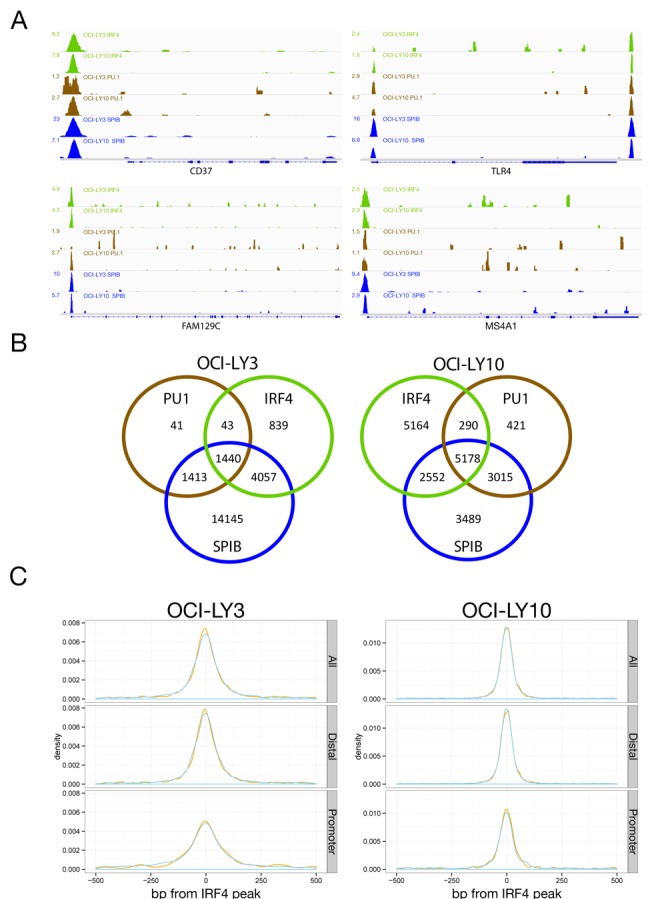
IRF4, SPIB and PU.1 distribution in ABC-DLBCL. (A) Representative examples of occupancy patterns for IRF4, PU.1 and SPIB in OCI-LY3 and OCI-LY10 cells are shown, normalized read-counts/million are indicated to the left of each track. (B) Venn diagrams showing the overlap of transcription factor cistromes for the indicated cell lines. (C) Density plots of the distribution of peak centres for SPIB (blue) and PU.1 (orange) relative to IRF4 peak centres. The *x*-axis shows 500 bp up- and down-stream of IRF4 peak centres at 0.

Occupancy of SPIB was confirmed at known targets including *BCL2A1, P2RY10*, *FCRL5*, *CD36*, *CD37* and *CD40* (Figure 1A and Supplementary Figure S2 and Table S1) (16,44–46). SPIB occupancy was identified at promoters of genes previously defined as PU.1 targets, such as *MS4A1* (*CD20*) (47), a critical element of the B-cell phenotype and target of therapeutic monoclonal antibody rituximab, and at promoters of several members of dispersed gene families, such as *TLR4*, *TLR7* and *TLR9*. SPIB binding was also identified across clustered gene families, such as *SP100*, *SP110* and *SP140*, and the *FCRL1–5* cluster. Important regulatory interactions for SPIB have been previously defined, first, in a positive feedback loop with the transcriptional factor *TCF4* (*E2–2*) during plasmacytoid dendritic cell ontogeny (48,49), and, second, in a negative feedback loop with *PRDM1* (*BLIMP1*) during plasma cell differentiation (13). Consistent with these regulatory interactions binding of SPIB to the previously identified PU.1/ETS-site within the *PRDM1* promoter (50), and binding to several elements within the *TCF4* gene was identified (Supplementary Figure S2). Thus, the overall pattern of occupancy detected for SPIB confirms known regulatory interactions, and provides to our knowledge the first genome-wide view of SPIB occupancy for the endogenous protein.

The cistromes of all three assessed transcription factors overlapped extensively between the two cell lines. Differences primarily derived from absolute numbers of binding events in each cell type, thus for IRF4 we observed 95% overlap of the LY3 cistrome among that of LY10, for PU.1 97% overlap of the LY3 cistrome among that of LY10 and for SPIB 91% overlap of the LY10 cistrome among that of LY3. As expected the cistromes of these factors were also highly interrelated within each cell line (Figure 1B). IRF4 occupancy occurred predominantly in the context of one or other ETS-partner, encompassing 87% of the IRF4 cistrome in OCI-LY3 and 61% in OCI-LY10. More than 95% of these sites were bound in the presence of SPIB in either cell line. Among sites occupied by ETS-factors without IRF4, PU.1 alone made a minor contribution. In contrast, occupancy by SPIB in the absence of PU.1 was a common feature (SPIB_Only 90% in LY3 and 50% in LY10; factor names separated by underscores are used to denote co-occupancy patterns in the remainder of the manuscript, e.g. IRF4 and SPIB co-occupancy = IRF4_SPIB). At co-occupied sites the peak centres showed a high degree of overlap and for the majority of IRF4 occupied sites the nearest peak centre for either SPIB or PU.1 was within 50 bp (Figure 1C). IRF4 occupancy in the absence of ETS-factors showed the largest bias towards promoters in both cell lines (34%), while other factor combinations showed lesser proportions of promoter occupancy (12.5–23%).

For comparison we additionally assessed the H929 myeloma cell line, which expresses high levels of PU.1 relative to SPIB (Supplementary Figure S1C). ChIP-seq from this cell line provides both an assessment of IRF4 occupancy in a distinct transcription factor context, and an additional control for the specificity of the SPIB antibody since the strong expression of PU.1 relative to SPIB would be expected to result in a reversal of the cistrome sizes in comparison to the two DLBCL cell lines. In the H929 cell line we identified 21946 PU.1, and 19755 IRF4 sites (Supplementary Figure S3A and B). In contrast, only 1193 SPIB sites were recovered which is consistent with the low level of protein expression. Of the IRF4 cistrome in H929 cells, 63.5% was occupied by IRF4 in the absence of PU.1 or SPIB, while 32.7% was occupied by IRF4 and PU.1. This represented a significant shift in favour of IRF4 occupancy in the absence of an ETS-factor partner in H929 relative to OCI-LY3 and LY10 (*p*-value = 2.2E–16 chi-squared test). SPIB alone or in conjunction with PU.1 made only a minor contribution to IRF4 occupancy in H929 myeloma cells.

### Occupancy confirms motif identity for SPIB and PU.1, and SPIB as predominant IRF4 partner at EICEs

**Figure 2.**
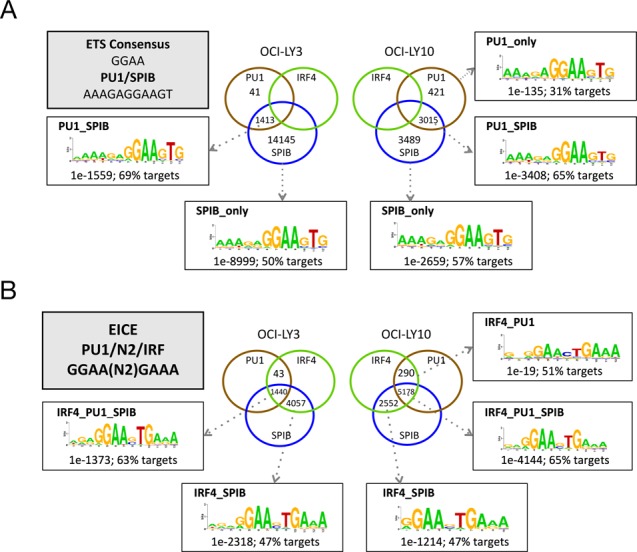
*De novo* motif discovery at SPIB and PU.1 occupied regions. Sequence motifs discovered by *de novo* motif detection with HOMER are shown, at regions occupied by (A) SPIB and/or PU.1 in the absence of IRF4, (B) SPIB and/or PU.1 in the presence of IRF4. Motifs are broken down by indicated co-occupancy pattern. Shown are top ranked motifs with enrichment and percentage of peak regions with motif match. The ETS, PU.1/SPIB and EICE consensus sequences are indicated for reference.

PU.1 and SPIB have at most subtle differences in preferred binding motif when assayed *in vitro* (8,51). Consistent with this *de novo* motifs identified in regions occupied by SPIB alone or in combination with PU.1, matched the previously defined consensus (Figure 2A and Supplementary Table S2) (52). Equally, EICEs were recovered from sites occupied by IRF4 and SPIB or PU.1 (Figure 2B) and at sites occupied by IRF4 and PU.1 in the H929 myeloma (Supplementary Figure S3C). The detection by *de novo* motif discovery of highly enriched motifs matching the *in vitro* determined consensus for SPIB, and the canonical EICE at co-occupied sites, provides further validation of the observed occupancy patterns. At IRF4_SPIB_PU.1 occupied sites a modestly increased frequency of additional EICE and ETS motifs (not overlapping with an adjacent EICE) was evident within 100 bp of the peak centre suggesting that some of these regions have the potential for combined occupancy by all three factors (Supplementary Figure S4). We conclude that in these ABC-DLBCL cell lines SPIB is the principle IRF4 partner at regulatory regions encompassing EICEs.

### SPIB regulates immune response genes

**Figure 3.**
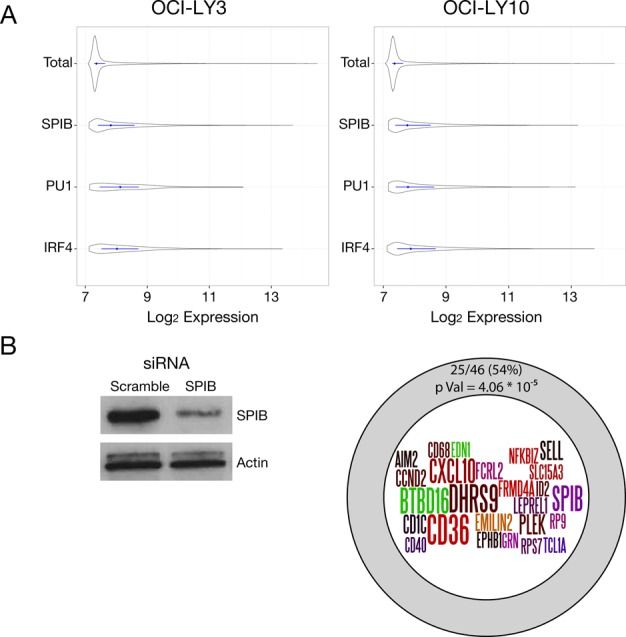
Gene expression associated with SPIB occupancy. (A) Gene expression values for OCI-LY3 (left panel) and LY10 (right panel) are shown as density plots, with the median and interquartile ranges (25th–75th) shown as a central line for all genes (Total) or genes associated with local factor occupancy (peak ±2kb of TSS) as shown on the *y*-axis, with log2 expression values on the *x*-axis. (B) Summary of SPIB knockdown results, representative western blot of SPIB knockdown in OCI-LY3 cells is shown on the left. The intersection of genes showing significant downregulation (adjusted *p*-value <0.05, >1.5-fold change) following 48 h SPIB knockdown with those also showing local SPIB occupancy (5 kb upstream/intragenic) is illustrated in the diagram on the right.

We next considered the relationship between local occupancy by SPIB, PU.1 and IRF4 (defined as a peak less than 5 kb upstream or within a gene body) and gene expression in each cell line (Figure 3A). The bulk of genes associated with local factor occupancy had low median expression values, but the distributions were as expected shifted towards positive gene expression in each cell line. Overall IRF4 associated genes showed the greatest shift towards higher median gene expression.

To further assess the relationship between SPIB occupancy and gene regulation we performed siRNA knockdown in OCI-LY3 and LY10 and assessed gene expression at 48 h (Figure 3B), SPIB knockdown was not as well sustained in OCI-LY10 and we therefore restricted analysis to OCI-LY3. Three hundred and six gene probes showed significant differences in expression on SPIB knockdown (False Discovery Rate (FDR) adjusted *p-*value < 0.05), corresponding to 283 genes (Supplementary Table S3). Of genes changing expression following SPIB knockdown 71/133 downregulated (*p-value* = 1.43E-10) and 68/150 upregulated (*p-value* = 1.5E-06) genes were linked to SPIB occupancy. Imposing a threshold of ≥1.5-fold change in expression restricted this to 88 genes changing expression, among which 25/46 downregulated (*p*-value = 4.06E-05) and 18/42 upregulated (*p*-value = 1.18E-02) genes were linked with local SPIB occupancy. This set of acutely responsive target genes, positively controlled by SPIB (Figure 3B), included the common elements of the ABC-DLBCL profile *CCND2* and *NFKBIZ* (1,14,24), as well as established (*SELL (Selectin-L/CD62L)*, *CD40*) and more recently defined (*FCRL2*) regulators of B-cell immune responses (53).

### BATF is an IRF4 co-factor in ABC-DLBCL cell lines

**Figure 4.**
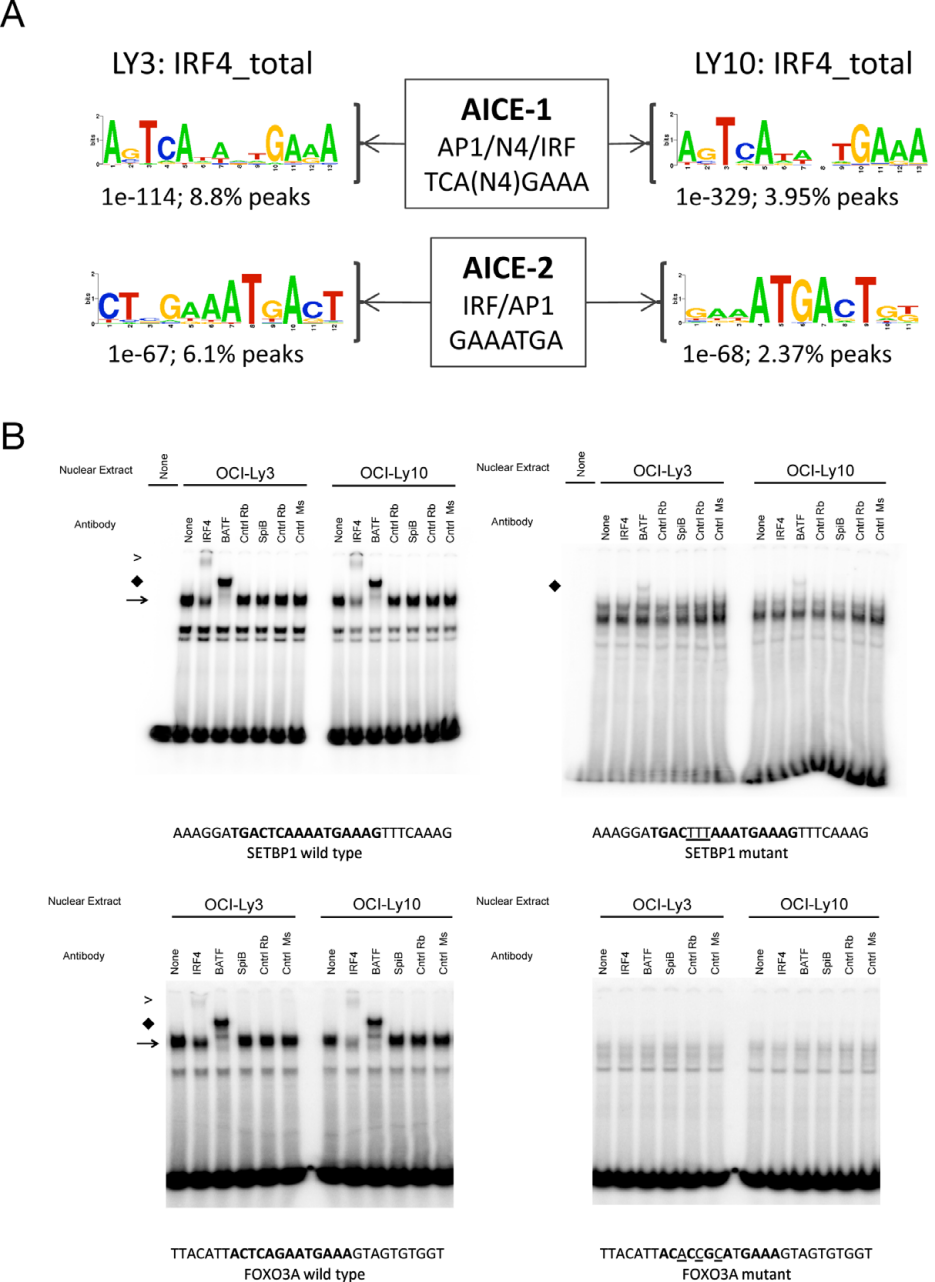
Association of BATF and IRF4 in ABC-DLBCL. (A) AICE motif variants identified by *de novo* motif detection among the complete IRF4 cistrome in OCI-LY3 and OCI-LY10 are shown as logos, along with the significance of enrichment and percent of peak regions with a motif match. (B) EMSA was performed with nuclear extracts of OCI-LY3 and LY10 cells using probes corresponding to wild type (left pairs of images) and mutated AICE motifs (right pairs of images) at IRF4 occupied sites in *SETBP1* (upper panels) and *FOXO3* (lower panels) genes. Binding partners were confirmed using indicated specific antibodies. Arrows identify primary BATF/IRF complex, and supershifted bands labelled ‘S’. Sequence of oligos used in EMSA with mutated bases where relevant are indicated below each panel.

While genomic occupancy in the presence of SPIB represented the predominant mode in both OCI-LY3 and LY10 cells, sites occupied by IRF4 in the absence of SPIB and PU.1 were of particular interest as these were likely to include regions bound by IRF4 in the context of additional cofactors, such as BATF. This component of the IRF4 cistrome included 839 peaks (13%) in OCI-LY3, but a greater proportion in OCI-LY10, 5164 peaks (39%) (Figure 1B). Notably, *de novo* motif detection either from the complete IRF4 cistrome from both ABC-DLBCL cell lines or the IRF4_Only peak subsets identified motifs matching AICEs (Figure 4A and Supplementary Figure S5A). These included both AICE-1 variants which place the AP1 component of the motif 5′ of the ‘GAAA’ sequence bound by IRFs with a 4-base spacing, and the AICE-2 variant in which the orientation is inverted and the core IRF site is immediately 5′ of the AP1 site.

We recently used a comparative analysis of gene expression across 10 DLBCL data sets to establish the genes most consistently associated with ABC- and GCB-DLBCL (24). BATF was among the 24 genes that were associated with the ABC-class in all data sets. We therefore performed EMSA to assess the potential for BATF to form DNA-binding complexes with IRF4 in the OCI-LY3 and LY10 cell lines. Probes encompassing AICEs associated with *SETBP1* and *FOXO3* genes both generated a dominant complex which was super-shifted by IRF4 or BATF antibodies (Figure 4B). As expected, mutation of the AP1 element of the consensus eliminated the formation of this cocomplex. A greater residual complex was observed on IRF4 antibody supershift, particularly in OCI-LY3 cells, and expression of IRF8 may provide an explanation for this observation as this factor can also form complexes with BATF at AICEs (20,21).

**Figure 5.**
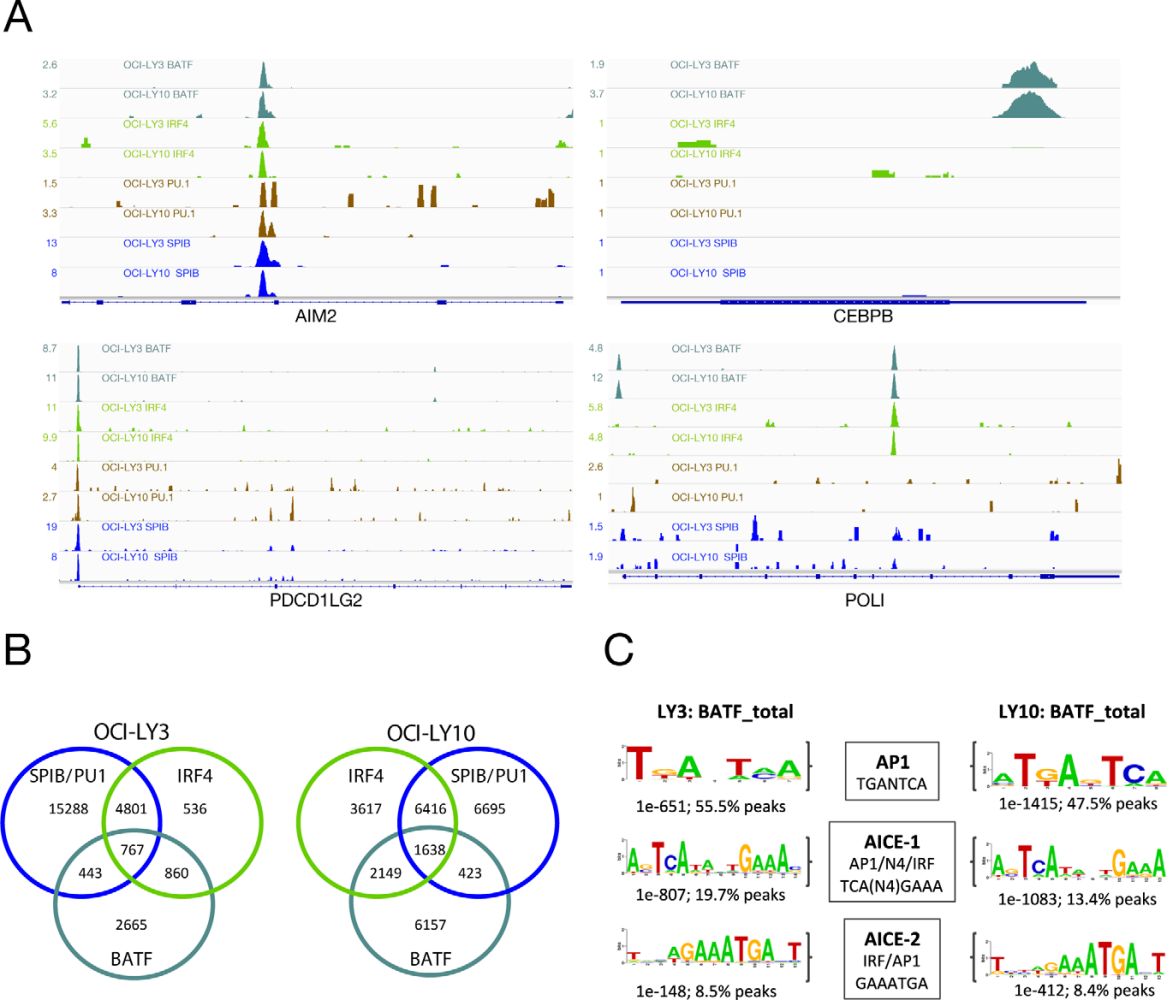
BATF occupancy in ABC-DLBCL. (A) Illustrates representative examples of BATF, IRF4, PU.1 and SPIB occupancy patterns in OCI-LY3 and OCI-LY10. (B) Venn diagram illustrating the extent of overlap between BATF, IRF4 and merged PU.1/SPIB cistromes in OCI-LY3 (left) and OCI-LY10 (right). (C) Representative motifs recovered from *de novo* motif detection for each cell line (OCI-LY3 left; OCI-LY10 right) illustrating the most common recovered motif of AP1 type, and AICE motif variants identified amongst the total BATF cistrome. Consensus sequences for AP1, AICE-1 and AICE-2 motifs are illustrated for reference.

**Figure 6.**
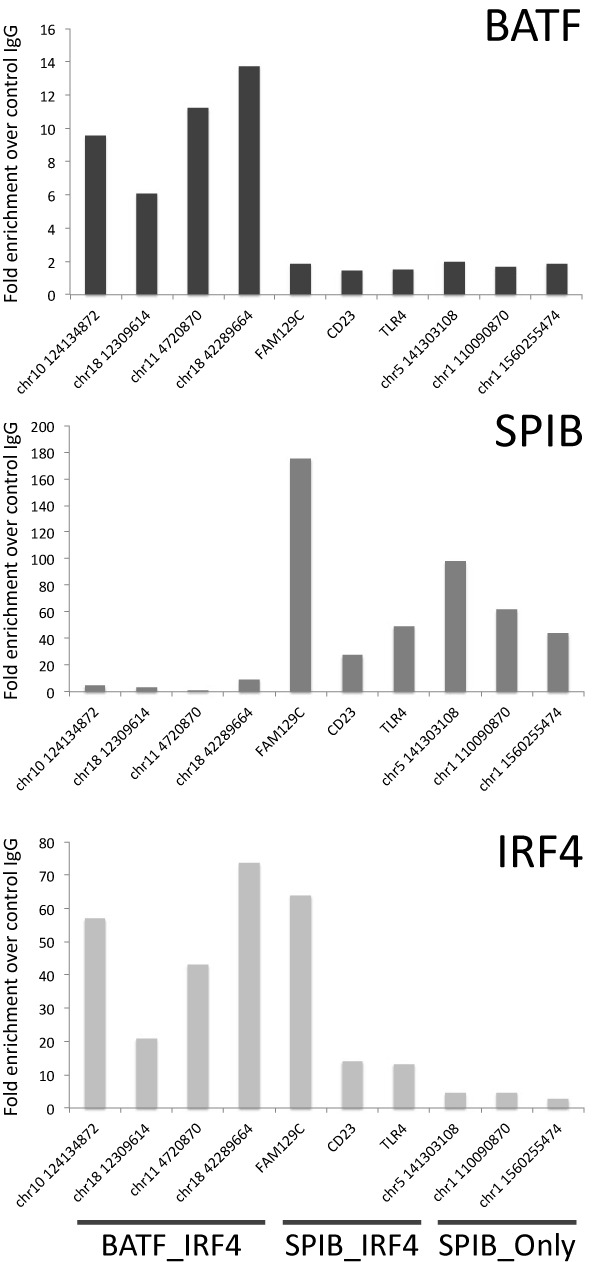
Differential occupancy patterns are confirmed by manual validation. Shown are representative results for BATF, SPIB and IRF4 ChIP using qPCR at selected targets representative of IRF4_BATF, IRF4_SPIB and SPIB_Only occupancy patterns as indicated at the bottom of the figure. Results are shown as fold enrichment relative to control IgG on the *y*-axis, promoter regions are indicated using official gene symbol, while other regulatory elements are indicated by genomic position (hg19) of the forward primer. Results are representative of two independent experiments from different chromatin batches derived from OCI-LY10 cells.

To assess the contribution of BATF to IRF4 binding more generally we performed ChIP-seq for BATF from both ABC-DLBCL cell lines (Figure 5A). This identified a total of 4735 and 10367 BATF peaks in OCI-LY3 and LY10, respectively (Supplementary Table S4). These overlapped with IRF4 peaks both in the absence and in the presence of SPIB and PU.1 (Figure 5B), while a lesser fraction of the BATF cistrome overlapped with SPIB or PU.1 in the absence of IRF4. Among peak regions occupied by BATF in both cell lines classical AP1 motifs and AICEs were identified as enriched in *de novo* motif discovery (Figure 5C). We manually validated a selection of 11 peak regions characterized by different occupancy patterns for IRF4, SPIB and BATF using ChIP-qPCR. These results verified the detection of different occupancy patterns (Figure 6).

The position of IRF4 and BATF peak summits at co-occupied sites showed similar distributions (Supplementary Figure S5B). To evaluate motif usage at peak regions occupied by IRF4 with SPIB, PU.1 and BATF (IRF4_SPIB-PU.1_BATF), or BATF alone (IRF4_BATF) we considered the 200 most significant peak regions. IRF4_BATF occupied regions were characterized by the expected AP1 and AICE motifs and rarely contained matches to either EICEs or ETS motifs. In contrast, IRF4_SPIB-PU.1_BATF occupied regions contained matches to EICE, ETS, AP1 and AICE motifs but few individual peak regions contained matches to all four motifs (Supplementary Figure S5C). Overall within this subset of multiply co-occupied peaks a higher frequency of EICEs was observed relative to AICEs indicating that SPIB is likely to provide the most common direct IRF4 cofactor at these multiply co-occupied sites in OCI-LY3 and LY10 cells.

### Distinct IRF4 occupancy patterns relate to cofactor positioning in Epstein–Barr virus (EBV) LCLs and ABC-DLBCL cell lines

**Figure 7.**
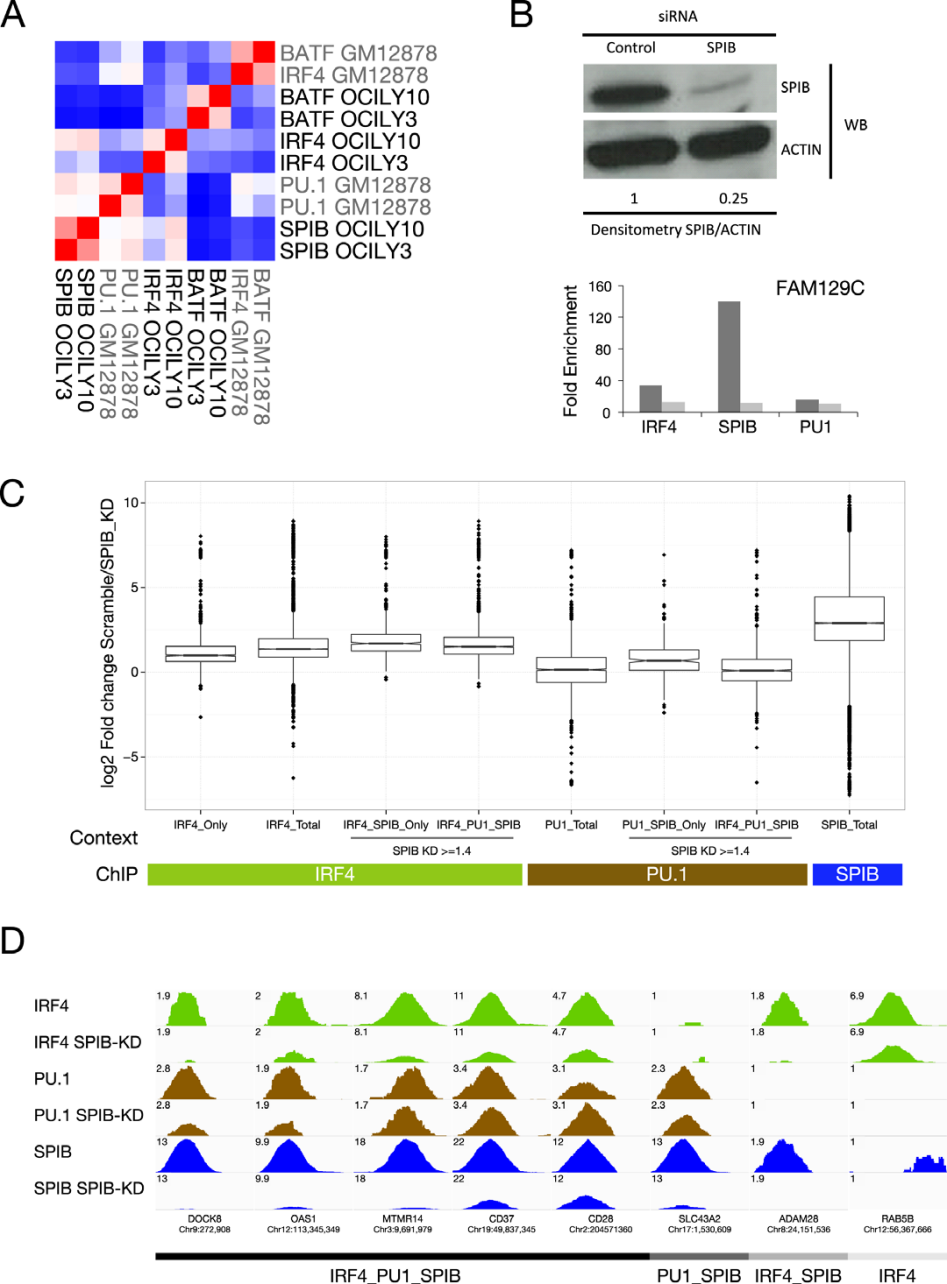
SPIB can act as a functionally dominant IRF4 partner in ABC-DLBCL. (A) IRF4 shows SPIB or BATF predominant genome-wide positioning in different contexts of post-germinal centre transformation. The genome-wide distribution of BATF, IRF4 and SPIB in OCI-LY3 and LY10 was compared to the distribution of BATF, IRF4 and PU.1 in ENCODE GM12878 data by Pearson's correlation. The pairwise correlations of occupancy determined at 100 bp resolution, are illustrated as a hierarchically clustered heat-map (blue = -0.2, white = 0.2 and red = 0.9 Pearson's correlation coefficient). (B) SPIB knockdown at 48 h was confirmed by western blot, and the degree of knockdown was confirmed by densitometry relative to ACTIN loading control, for cells used in ChIP-seq analysis. The effect of knockdown, in matching chromatin, was evaluated by qPCR at a positive control regulatory element in the *FAM129C* promoter for IRF4, SPIB and PU.1 ChIP samples. Results are shown as fold enrichment over control IgG. (C) Illustrates the genome-wide analysis of the impact of SPIB knockdown shown in (B) on the occupancy of IRF4, PU.1 and SPIB indicated in coloured bars beneath graph, at regulatory elements divided according to the occupancy context shown as *x*-axis labels. The box and whisker plots display the log_2_ fold change in factor occupancy between control siRNA and SPIB siRNA treated OCI-LY3 cells, across all merged regulatory elements of the indicated occupancy type. Significances are indicated in the text, only the comparison of PU.1_total versus IRF4_PU.1_SPIB were not significantly different, all other comparison are significant at *p-*value ≤ 7.7E-04. (D) Illustrates examples of IRF4, PU.1 and SPIB occupancy in OCI-LY3 cells in control and SPIB siRNA treated OCI-LY3 cells. Changes in peak height can be compared for each transcription factor by setting the visualized reads/million to the same maximum value observed for each transcription factor at the indicated region.

Signalling via the LMP1 and LMP2A proteins plays a critical role in EBV lymphoblastoid transformation (54). These viral proteins provide mimics of constitutive CD40 and B-cell receptor signalling, corresponding to two critical pathways of oncogene activation in ABC-DLBCL (2). Lymphoblastoid cell lines, and in particular the ENCODE data derived from GM12878 LCLs (55), thus provide the opportunity for relevant comparison to ABC-DLBCL. We therefore assessed the IRF4, BATF and SPIB cistrome in OCI-LY3 and LY10 cells, selecting the dominant ETS factor for balanced data set number, against the IRF4, BATF and PU.1 cistromes in ENCODE GM12878 data (55). The distributions of SPIB binding in the LY3 and LY10 cell lines correlated most significantly with that of PU.1 in GM12878, while IRF4 binding in LY3 and LY10 cell lines correlated most highly with that of SPIB, and weakly with the distribution of PU.1 in GM12878 (Figure 7A). In contrast, the IRF4 distribution in GM12878 correlated with BATF and to a lesser extent with PU.1 in the matching cell line (56). Thus, the overall positioning of IRF4 in two related contexts of post-germinal centre B-cell transformation shows distinct linkage to either SPIB/PU.1 or BATF centred distributions.

### SPIB is the functionally dominant IRF4 co-factor in the OCI-LY3 ABC-DLBCL cell line

The overall frequency of IRF4 and SPIB co-occupancy, and the genome-wide correlation analysis supported a dominant role for SPIB in determining IRF4 occupancy in the ABC-DLBCL cell lines. However, the extent to which SPIB provided an essential determinant of IRF4 DNA-binding in this context was uncertain; to address this question, we knocked-down SPIB expression in OCI-LY3 cells, to a degree sufficient to impact on both SPIB and IRF4 binding at a selected positive control site (Figure 7B), and performed ChIP-seq for SPIB, PU.1 and IRF4. Overall we observed a highly significant loss of SPIB but not PU.1 occupancy genome-wide, further validating the specificity of our SPIB ChIP-seq data (Figure 7B and C). Although a subset of regulatory elements did show a reciprocal increase in PU.1 occupancy on SPIB depletion, such as a regulatory element near CD28 shown in Figure 7D, generally PU.1 did not compensate for SPIB depletion by increased binding (Figure 7C). In contrast, depletion of SPIB was accompanied by a genome-wide loss of IRF4 occupancy. Even among those sites bound by IRF4 in the absence of either ETS-factor partner an overall loss of IRF4 occupancy was observed, which contrasted with the absence of any impact on *IRF4* mRNA expression on SPIB knockdown (Figure 7C IRF4_Only and Supplementary Table S3). However, using the set of regulatory elements bound by IRF4 in the absence of either PU.1 or SPIB for comparison (IRF4_Only), the loss of IRF4 binding on SPIB knockdown was significantly greater at sites co-occupied by IRF4 in the presence of SPIB irrespective of PU.1 co-occupancy (IRF4_SPIB_Only, *p*-value = 6.85E-28; IRF4_SPIB_PU.1, *p*-value = 5.47E-26). Furthermore, although genome-wide occupancy by IRF4 was responsive to loss of SPIB, a small fraction (∼8%) of all IRF4 occupied sites was unaffected by SPIB depletion (fold-change <1.4). This stable subset of IRF4 occupied sites was significantly enriched for regulatory elements bound by IRF4 in the absence of SPIB (43%, *p*-value = 1.23E-29). Thus, in the OCI-LY3 ABC-DLBCL cell line, PU.1 fails to compensate acutely for SPIB depletion and a general-shift towards an alternate pattern of BATF-centred IRF4 occupancy is not observed. However, while IRF4 occupancy is globally responsive to SPIB knockdown, those sites occupied by IRF4 in the absence of SPIB are as expected most resilient. We conclude that in this context SPIB provides the functionally dominant determinant of IRF4 genomic occupancy, and neither PU.1 nor BATF acutely compensate to maintain IRF4 occupancy or drive redistribution of IRF4 to a different occupancy pattern.

### SPIB occupancy is linked to genes overexpressed in primary ABC-DLBCL with high *SPIB* and low *BATF* expression

The intensity of SPIB expression has been linked to genomic amplification or translocation of chr19 in ABC-DLBCL (14), and SPIB shows a less consistent differential expression between GCB- and ABC-DLBCL than BATF (24). We reasoned, therefore, that the relative expression of BATF and SPIB might contribute to heterogeneity of tumour biology among ABC-DLBCL.

Although the classification of DLBCL into cell of origin classes has provided a central framework for understanding this disease, a number of different algorithms have been used to implement the classification in different studies. We recently described a detailed evaluation of classifier algorithms across a range of available DLBCL gene expression data sets (24). We established a robust platform independent classifier tool, the DLBCL Automatic Classifier (DAC), which allows consistent classification of multiple data sets and is effective on data generated both from fresh frozen and formalin-fixed paraffin embedded samples. With this tool we previously performed a meta-analysis of gene expression across 10 uniformly classified DLBCL gene expression data sets (24). To address whether the relative expression of BATF and SPIB might contribute to disease heterogeneity, we first examined the pairwise correlation of these transcription factors in ABC-DLBCL using the cases contained in the 10 publically available data sets and the classifications of these cases that we have previously established using DAC (24). In this analysis, one data set (GSE19246) emerged as a consistent outlier and was therefore excluded from further assessment. Each transcription factor pairing showed evidence of a modest positive correlation; however, overall there was a greater positive correlation of *IRF4* and *BATF* (average Spearmann's correlation = 0.53 ± 0.08) than *SPIB* and *BATF* (average Spearmann's correlation = 0.40 ± 0.09) or *IRF4* and *SPIB* (average Spearmann's correlation = 0.42 ± 0.09) (Supplementary Figure S6).

We reasoned that since SPIB and BATF provide distinct regulatory information, the observed variability in *BATF* and *SPIB* mRNA expression in ABC-DLBCL might be associated with differences in disease biology. To address this we separated the ABC-DLBCL cases into four groups, by using a contingency table approach divided by high and/or low expression of *BATF* and *SPIB* mRNA (top and bottom 50% of expression as threshold). We then determined differential gene expression (*p*-value < 0.05) between ABC-DLBCL cases characterized by the two extremes of high-*SPIB* and low-*BATF* versus high-*BATF* and low-*SPIB* mRNA expression in each data set. We subsequently refer to these subgroups as SPIB^high^/BATF^low^-ABC-DLBCL and SPIB^low^/BATF^high^-ABC-DLBCL. To identify genes consistently associated with either of these two extremes we used a threshold of differential expression in four or more data sets, and refer to the resulting lists as ‘meta-profiles’. In this pairwise comparison a total of 198 genes were identified as overexpressed in SPIB^high^/BATF^low^-ABC-DLBCL and 237 genes in SPIB^low^/BATF^high^-ABC-DLBCL (Supplementary Table S5).

**Figure 8.**
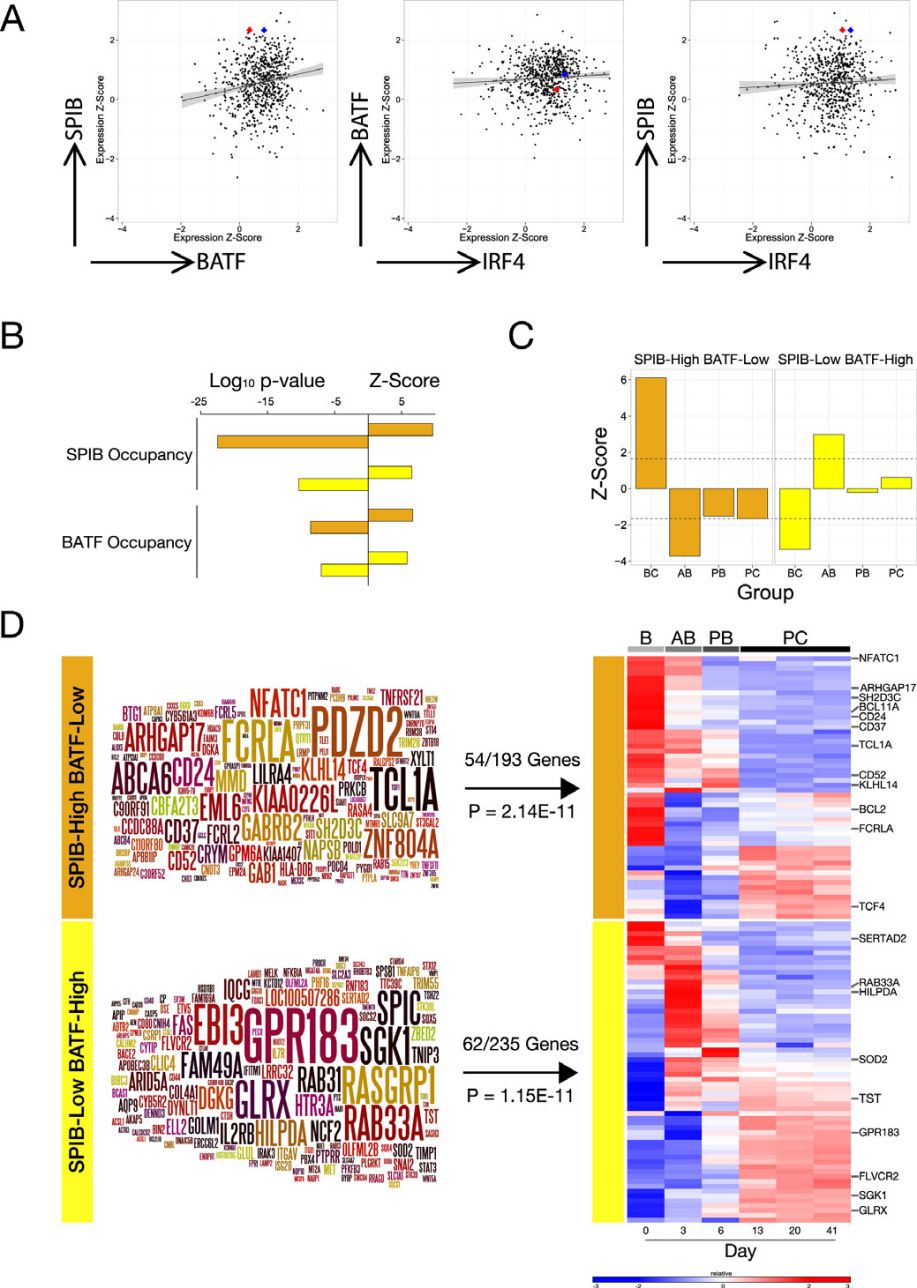
SPIB^high^/BATF^low^ and SPIB^low^/BATF^high^-ABC-DLBCL are differentially associated with transcription factor occupancy and stages of B-cell differentiation. (A) The normalized expression values for *SPIB*, *BATF* and *IRF4* mRNA in OCI-LY3 and LY10 cells are shown relative to expression of these factors in primary ABC-DLBCL across 9 gene expression data sets. The scatter plots illustrate pairwise comparisons as indicated by arrow labels, *x*- and *y*-axis represent normalized expression values as *z*-scores. The correlation is indicated as a line with 95% confidence interval as shading, and the expression values for OCI-LY3 and OCI-LY10 are shown as red and blue spots, respectively. (B) The enrichment of genes (hypergeometric test) with local SPIB and BATF occupancy (−5 Kb or intragenic) in OCI-LY3 and LY10 is shown, as indicated on the left of the bar graph, among the meta-profiles for SPIB^high^/BATF^low^ (orange bars) and SPIB^low^/BATF^high^-ABC-DLBCL (yellow bars). The bar graph illustrates the Log_10_*p*-value to the left and *Z*-score to the right on the *x*-axis. (C) The enrichment or depletion of genes maximally expressed at different stages of *in vitro* human B-cell differentiation to the plasma cell stage, among SPIB^high^/BATF^low^ (orange bars) and SPIB^low^/BATF^high^-ABC-DLBCL (yellow bars) meta-profiles was determined using a bootstrapping approach (1E7 randomizations). Shown are *z*-scores on the *y*-axis relating to categories following the order B-cell (BC), activated B-cell (AB), plasmablast (PB) and plasma cell (PC) as indicated from left to right. The dotted line represents *p*-value of 0.05 (D) The SPIB^high^/BATF^low^ (orange bar) and SPIB^low^/BATF^high^-ABC-DLBCL (yellow bar) meta-profiles are shown as Wordles with the degree and consistency of differential expression represented by font size, also indicated is the enrichment of meta-profile genes among genes showing dynamic expression during B-cell terminal differentiation in the heat-map (blue to red colour scale representing low to high mRNA expression) to the right.

As an approximate assessment of the relationship between *SPIB*, *BATF* and *IRF4* expression in OCI-LY3 and LY10 cell lines and primary ABC-DLBCLs we superimposed the normalized expression values for these cell lines onto the distributions derived for primary ABC-DLBCL across all data sets. With the caveat that gene expression assessments from primary tumour samples derive from mixed cell types, this confirmed that OCI-LY3 and LY10 fell within the general distribution of expression values for *BATF* and *IRF4*, and at the high end of the *SPIB* distribution (Figure 8A). We then assessed the overlap between local SPIB and BATF occupancy in OCI-LY3 and LY10 cells and the meta-profiles of SPIB^high^/BATF^low^ and SPIB^low^/BATF^high^-ABC-DLBCL (Figure 8B). Genes with occupancy by SPIB or BATF, within the gene body or 5 kb upstream, were significantly enriched among both SPIB^high^/BATF^low^ and SPIB^low^/BATF^high^-ABC-DLBCL meta-profiles. However, SPIB occupancy showed a substantially more significant enrichment in the SPIB^high^/BATF^low^ meta-profile (*p*-value = 3.43E-23), than the SPIB^low^/BATF^high^ meta-profile (*p*-value = 4.47E-11). In contrast, BATF occupancy showed only a minor difference in enrichment between the two meta-profiles (SPIB^high^/BATF^low^ meta-profile *p*-value = 2.62E-09 versus SPIB^low^/BATF^high^ meta-profile *p*-value = 9.77E-08). This supports a direct regulatory contribution by SPIB to preferential gene expression in primary SPIB^high^/BATF^low^-ABC-DLBCL.

### SPIB^high^/BATF^low^ and SPIB^low^/BATF^high^-ABC-DLBCL are reciprocally linked to distinct stages of B-cell differentiation

The biology of ABC-DLBCL is related to cells trapped in abortive plasma cell differentiation. We noted that during *in vitro* B-cell differentiation to plasma cells, *BATF* expression was induced in activated B-cells prior to the loss of B-cell phenotype, while *SPIB* is modestly reduced in activated B-cells and repressed upon transition to plasmablasts (Supplementary Figure S7). We, therefore, considered that the differences in gene expression linked to the subgroups of ABC-DLBCL defined by relative SPIB and BATF expression might also relate to different stages of B-cell to plasma cell differentiation. To address this we intersected the meta-profiles for SPIB^high^/BATF^low^ and SPIB^low^/BATF^high^-ABC-DLBCL with gene expression data, derived from an *in vitro* model we have recently developed, spanning the *in vitro* differentiation of resting human B-cells to long-lived plasma cells (28). Notably, both meta-profiles were significantly enriched for genes showing dynamic expression changes during B-cell to plasma cell differentiation (54/193 SPIB^high^/BATF^low^ *p*-value = 2.14E-11, 62/235 SPIB^low^/BATF^high^ *p*-value = 1.15E-11). Furthermore, the meta-profiles were also skewed relative to the B-cell differentiation time course (Figure 8C and D). SPIB^high^/BATF^low^-ABC-DLBCL was positively associated with genes with maximal expression in B-cells (*Z*-score = +6.1, *p*-value = 0) and significantly depleted of genes expressed at later stages of differentiation in particular the *in vitro* activated B-cell state (AB genes *Z*-score = −3.7, *p*-value = 2.4E-06). In contrast, SPIB^low^/BATF^high^ ABC-DLBCL showed a reciprocal pattern of association with significant enrichment of *in vitro* activated B-cell genes (*Z*-score = 2.97, *p*-value = 0.003) and significant depletion of genes expressed in resting B-cells (*Z*-score = −3.33, *p*-value = 7.05E-05). However, there was no significant difference, between the SPIB^high^/BATF^low^ and SPIB^low^/BATF^high^-ABC-DLBCL subgroups, in the expression of classifier genes used to establish the ABC versus GCB-DLBCL classification or the DAC-classification confidence, which provides an overall assessment for each case of the likelihood of belonging to one of the classes of the cell of origin classification (Supplementary Table S6). We conclude that these two subgroups show similar expression of the principle classifier genes used to identify ABC-DLBCL but differ in their relationship to stages of B-cell differentiation: the SPIB^high^/BATF^low^-ABC-DLBCL subgroup is characterized by preferential retention of genes expressed in resting B-cells, while SPIB^low^/BATF^high^-ABC-DLBCL displays a more exaggerated similarity to *in vitro* activated B-cells.

### SPIB^high^/BATF^low^ and SPIB^low^/BATF^high^ ABC-DLBCL are linked to distinct gene sets

In order to gain further insight into the potential relationships of SPIB^high^/BATF^low^ and SPIB^low^/BATF^high^-ABC-DLBCL and previously defined molecular pathways, we performed an analysis of gene signature enrichment using a hypergeometric test. The extensive compendium of gene signatures tested were derived from GeneSigDB, MSigDB, Staudt, Shipp and Du laboratories, and were filtered for gene signatures of less than 1000 genes in size (13978 in total) (34,57–60). After correction for false discovery there remained an extensive list of enriched signatures for both meta-profiles. At FDR corrected *p*-value < 0.001, 109 and 281 gene signatures overlapped significantly with the SPIB^high^/BATF^low^ and the SPIB^low^/BATF^high^ meta-profiles, respectively (Supplementary Table S7). The SPIB^high^/BATF^low^ meta-profile showed most significant overlap with signatures of the resting B-cell state (e.g. Pan_B_U133plus, FDR *p*-value = 2.37E-15, Blood_Module-1.3_B_cells, FDR *p*-value = 1.72E-11), but also showed enrichment of signatures related to the activated B-cell lymphoma class (e.g. ABCgtGCB_U133AB, FDR *p*-value = 1.24E-09) as well as several signatures related to plasmacytoid dendritic cells (e.g. Dendritic_cell_CD123pos_blood, FDR *p*-value = 2.21E-10; GSE29618_PDC_VS_MDC_UP, FDR *p*-value = 4.13E-10). In relation to SPIB itself, the SPIB^high^/BATF^low^ meta-profile was notably enriched both for an external signature related to evolutionarily conserved PU.1/SPIB motifs in gene promoters (MSigDB_C3 signature RGAGGAARY_V$PU1_Q6, FDR *p*-value = 9.42E-06) and importantly also for expression of genes in chr19q13 (MSigDB_C1 signature chr19q13, FDR *p*-value = 8.38E-05), the cytoband containing the *SPIB* gene, while no other cytoband showed enrichment at FDR corrected *p*-value < 0.001 for either meta-profile. Interestingly, when considering genes 2Mb either side of the *SPIB* transcriptional start site, enrichment was exclusively observed for genes upstream/centromeric to *SPIB* (Supplementary Table S7). Coexpression of genes associated with chr19 amplification, as described by Lenz *et al.* (14), in the SPIB^high^/BATF^low^ meta-profile suggests that such amplification is a contributing factor to the pathogenesis of this subgroup identifiable from expression profiles.

In contrast, the SPIB^low^/BATF^high^ meta-profile was most significantly enriched for genes associated with STAT3 activation in ABC-DLBCL (STAT3high_ABC_DLBCL_subgroup, FDR *p*-value = 2.85E-17), while among other signaling pathway signatures those linked to nuclear factor kappa-light-chain-enhancer of activated B cells (NFκB) in ABC-DLBCL were also significantly enriched (e.g. NFKB_UP_BCR_paper, FDR *p*-value = 2.22E-08; BASSO_CD40_SIGNALING_UP, FDR *p*-value = 6E-05) (Supplementary Table S7). This is also consistent with the enrichment of genes at the activated B-cell stage of *in vitro* differentiation (Figure 8D), since this is driven by addition of IL21, a potent STAT3 activator, and CD40 ligation. However, it is worth noting that the SPIB^low^/BATF^high^ meta-profile does not overlap with all STAT3 related signatures to a similar degree. Genes included in a recent expression-based signature of STAT3 activation, in DLBCL as a whole (61), showed no significant enrichment in the SPIB^low^/BATF^high^ meta-profile (HUANG_PY_STAT3_Total, overlap 2/32, FDR *p*-value = 0.2, or HUANG_PY_STAT3_11Sig, overlap 0/11 genes, FDR *p*-value = 0.95). We conclude that separating the ABC-DLBCL subset according to relative expression of the two IRF4 cofactors SPIB and BATF identifies subgroups differentially linked to previously defined features of DLBCL tumour biology.

### High SPIB expression is linked to a group of ABC-DLBCL with good clinical outcome

**Figure 9.**
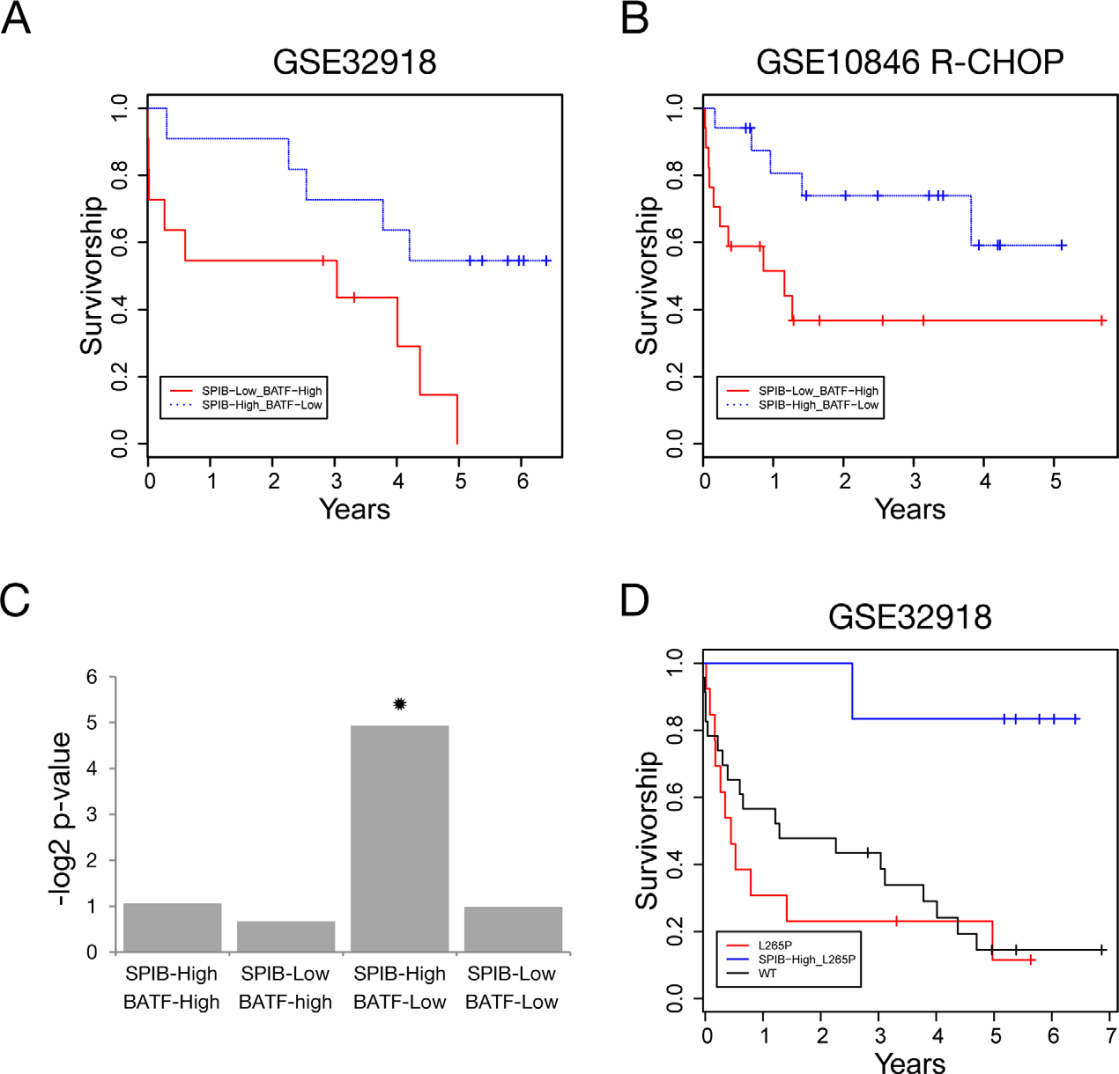
SPIB and BATF expression is linked to outcome and MYD88 mutation status in ABC-DLBCL. (A) Shows Kaplan–Meier analysis for overall survival of SPIB^high^/BATF^low^ (blue line), compared to SPIB^low^/BATF^high^-ABC-DLBCL (red line) cases in data set GSE32918. (B) Displays the Kaplan–Meier analysis for overall survival of SPIB^high^/BATF^low^ (blue line), compared to SPIB^low^/BATF^high^-ABC-DLBCL (red line) cases in data set GSE10846 R-CHOP component, using cases classified as ABC-DLBCL with our implementation of the cell of origin classification, DAC. (C) Illustrates the enrichment of *MYD88*-L265P mutations among the four ABC-DLCBL subgroups defined by high/low *SPIB* and high/low *BATF* mRNA expression. The *y*-axis represents the log_2_ of the *p*-value of enrichment. Significant enrichment is only observed in the SPIB^high^/BATF^low^ subgroup indicated by a star. (D) Illustrates Kaplain–Meier analysis of overall survival for ABC-DLBCL cases in data set GSE32918, divided according to *MYD88* mutation status, wild type (black line), *MYD88*-L265P mutation and SPIB^high^/BATF^low^ expression profile, or all other *MYD88*-L265P mutated ABC-DLBCL cases (red line). *MYD88* mutations other than L265P, detected by Sanger sequencing, of which there were three cases were included in the ‘wild type’ category to reflect a clinical scenario of targeted *MYD88*-L265P mutation detection.

We noted that the SPIB^high^/BATF^low^-ABC-DLBCL subgroup in our U.K. population-based patient cohort of DLBCL treated with R-CHOP chemotherapy (62), (GSE32918), was characterized by a relatively good survival and included a subgroup of patients with survival beyond 5 years (Figure 9A). Since case numbers were limiting we next asked whether a similar association between survival and differential *SPIB* and *BATF* expression could be observed in other data sets of cases treated with similar immunochemotherapy. We first examined this association in the data set GSE10846, generated by the Lymphoma Leukemia Molecular Profiling Project (LLMPP), which is the largest available data set derived from fresh frozen, rather than formalin-fixed paraffin embedded material, and has provided a reference data set for the association between gene expression profiles and clinical outcome in DLBCL in the era of immunochemotherapy (29). In this data set SPIB^high^/BATF^low^-ABC-DLBCL was similarly characterized by a good outcome (Figure 9B).

Since the choice of algorithm used to implement the cell of origin classification does affect the classification of a subset of cases with marginal expression values for classifier genes (24), we also addressed whether the association of outcome with the differential *SPIB/BATF* expression was affected by the use of our implementation of the cell of origin classifier. However, this was not the case since a similar good outcome was observed for SPIB^high^/BATF^low^-ABC-DLBCL cases when using the pre-assigned classes for GSE10846 in the Gene Expression Omnibus (Supplementary Figure S8). This was consistent with the fact that the assignments of DLBCL to cell of origin classes differ for only a minority of cases overall between our classifications and those previously assigned to cases in GSE10846 (24).

In contrast to the concordant results observed between the LLMPP data, GSE10846 (29), and our U.K. population-based data, GSE32918 (62), when we examined a separate data set of R-CHOP treated DLBCL cases generated by The International DLBCL Rituximab-CHOP Consortium Program (GSE31312) (37), we found no significant difference in survival between SPIB^high^/BATF^low^ and SPIB^low^/BATF^high^-ABC-DLBCL. Nonetheless, the concordant results observed in GSE10846 and GSE32918 indicate that high *SPIB* and low *BATF* expression can identify a subgroup of ABC-DLBCL cases with good outcome, which in the context of our U.K. population-based cohort includes a small subset of ABC-DLBCL cases treated with R-CHOP displaying 5 year or greater survival.

### SPIB^high^/BATF^low^-ABC-DLBCL is linked to MYD88 mutation status and the combination identifies a group with distinct favourable outcome

*MYD88* mutation is an oncogenic event strongly associated with ABC-DLBCL and Waldenstrom macroglobulinemia, the recurrent L265P mutation of MYD88 accounts for the majority of this association (63,64). MYD88 is a principle signal transduction component downstream of TLRs and IL1R. A functional linkage between SPIB expression and TLR/MYD88 pathway activation has been identified by Yang *et al.* in which SPIB represses autocrine IFN secretion allowing ABC-DLBCL survival in the context of MYD88 mutation (16). At the same time, while the presence of a MYD88 mutation may promote receptor-independent signalling (63), TLR activation also contributes to signal transduction in the context of MYD88-L265P in ABC-DLBCL and in B-cells engineered to express mutant MYD88 (65,66). The *MYD88*-L265P mutation is present in both OCI-LY3 and LY10 cells (63), and we noted that SPIB binding was present in the promoters of *TLR4*, *7* and *9* and in the vicinity of *MYD88* itself in these cells. We, therefore, asked whether an association between *SPIB* expression and *MYD88* mutation might also be observed in primary ABC-DLBCL. We examined the *MYD88* mutation status of ABC-DLBCL cases in our cohort (62), and found a statistically significant association between cases with high *SPIB* and low *BATF* expression and the presence of a *MYD88* mutation in general (7/9 cases, *p*-value = 0.015), or the common *MYD88*-L265P mutation in particular (6/9 cases, *p*-value = 0.03) (Figure 9C and Supplementary Table S8). In contrast, there was no significant association between *MYD88* mutation status and any of the other combinations of *SPIB* and *BATF* expression, among which *MYD88* mutations were randomly distributed. Notably, among the six SPIB^high^/BATF^low^-ABC-DLBCL patients with *MYD88* mutations all but one survived for 5 years or more, the patient who died during follow-up was an 85-year-old who survived for 2.5 years but had not received treatment with curative intent. Thus, the identification of SPIB^high^/BATF^low^ mRNA expression may provide a tool to identify a subset of ABC-DLBCL patients with *MYD88*-L265P mutation who have a good response to current immunochemotherapy.

## DISCUSSION

IRF4 is at the centre of both the transcriptional program of B-cell terminal differentiation and of ABC-DLBCL. IRF4 engages in cooperative interactions with different transcription factor partners at distinct DNA elements (5,67), providing the basis for varied transcriptional input across cell states. Our findings suggest that the balance of IRF4 partner expression between SPIB and BATF identifies distinct subgroups of ABC-DLBCL linked to different stages of B-cell differentiation, oncogenic pathway activation and clinical outcome.

While several potential IRF4 transcription factor partners have been described (5), recent genome-wide studies have so far identified three predominant modes of IRF4 DNA-binding: at EICEs (16), at AICEs (18–20) and at repeats of the IRF ‘GAAA’ core consensus matching the ISRE pattern (18,68). A shift in favour of IRF4 binding at ISRE-like sequences has recently been identified as a transition point during plasma cell differentiation (69). These modes of DNA-binding by IRF4 are not mutually exclusive. In the OCI-LY3 and LY10 ABC-DLBCL cell lines, motif enrichment provided evidence for all three patterns of occupancy with EICEs predominating over co-occupancy with BATF at AICEs or occupancy at ISRE containing sequences. In the myeloma cell line H929, IRF4 occupancy in the context of AICEs was not observed, which is most likely to reflect absent or low BATF expression. While PU.1 expression in H929 cells resulted in frequent occupancy of IRF4 at EICEs, nonetheless IRF4 occupancy in the absence of PU.1 at regulatory elements characterized by ISRE or simple ‘GAAA’ core elements was most frequent. Of note, PU.1 expression is not uniform across primary myelomas or myeloma cell lines, suggesting the potential for significant variation in transcriptional input from IRF4 in plasma cell malignancies, which will be important to explore in future.

Frequent IRF4 occupancy at regulatory elements containing EICEs was recently described in the HBL1 ABC-DLBCL cell line by Yang *et al.* (16). This study additionally examined SPIB occupancy, and thus identified a central role for IRF4_SPIB heterodimers in several aspects of ABC-DLBCL biology. However, the use of HBL1 cells engineered to express biotin-tagged SPIB meant that the relative contribution of endogenous SPIB or PU.1 to IRF4 occupancy was not established. Here, we have addressed this issue and our data put the role of IRF4_SPIB heterodimers in ABC-DLBCL in a new context. In cell lines with strong SPIB expression this association is both quantitatively and functionally dominant, as shown by the fact that SPIB knockdown in the OCI-LY3 cell line leads to extensive loss of IRF4 DNA-binding, without compensation by PU.1 or redistribution of IRF4 to a more BATF-centred distribution. However, our data also show that input from SPIB is modified by a significant contribution from BATF both in the context of combined occupancy of regulatory elements by BATF, IRF4 and SPIB and as an independent IRF4 cofactor.

Comparison to ENCODE data demonstrates that the pattern of IRF4 occupancy in ABC-DLBCLs differs significantly from that in the EBV lymphoblastoid cell line GM12878. In the latter cell line a dominant contribution is made by BATF to IRF4 occupancy and BATF emerges as more highly correlated with IRF4 than PU.1 genome-wide (56). Thus, the GM12878 LCL provides a contrasting instance in which BATF is the predominant IRF4 partner in a transformed post-germinal centre B-cell. Interestingly, EBV-derived transcription factors expressed in LCLs have been recently shown to extensively cooccupy regulatory elements bound by BATF, IRF4 and PU.1 (70,71), and BATF has been previously identified as a direct target induced by EBNA2 (72). It will therefore be important to establish whether a BATF-centred IRF4 distribution can occur during B-cell activation or in primary SPIB^low^/BATF^high^-ABC-DLBCLs, or whether this pattern of occupancy observed in GM12878 cells reflects a regulatory state specific to EBV transformation. If so this would be predicted to pertain as the predominant mode of IRF4 occupancy in EBV-driven B-cell malignancies, such as EBV-associated DLBCL and EBV-associated classical Hodgkin lymphoma. Together the data are consistent with a general model of context-dependent IRF4 activity, and indicate that IRF4 expression in post-germinal centre B-cells malignancies can be linked to several quite distinct transcriptional states.

It is inevitable that a disease category, such as ABC-DLBCL, encompasses heterogeneity, but this is particularly relevant where one of the principle classifier genes used to establish the category encodes a transcription factor that can display the wide range of cis-regulatory occupancy observed for IRF4. The interest of analysing the nature of this heterogeneity lies on the one hand in identifying significant differences in clinical outcome and on the other in what such heterogeneity indicates in relation to disease biology. The biological validity of subdividing ABC-DLBCL based on *SPIB* and *BATF* expression is supported by the reciprocal association of the resulting subgroups with genes linked to distinct stages of B-cell differentiation. That SPIB^high^/BATF^low^-ABC-DLBCL is more significantly associated with genes expressed in resting B-cells is generally consistent with what is known of the normal function and expression pattern of SPIB which has previously been identified as a regulator of B-cell signaling pathways and a repressor of plasma cell differentiation (10–13). Furthermore, the significant enrichment of genes on chr19 in the vicinity of SPIB, including genes previously identified as coordinately overexpressed in ABC-DLBCLs with amplification of chr19 by Lenz *et al.* (14), is consistent with chr19 amplification providing a pathogenetic mechanism in the SPIB^high^/BATF^low^-ABC-DLBCL subgroup. In contrast, the enrichment in the SPIB^low^/BATF^high^-ABC-DLBCL meta-profile of genes expressed in B-cells following IL21 and CD40L activation, STAT3-high ABC-DLBCL and NFκB and CD40 activation, points to combined activation of the STAT3 and CD40/NFκB pathways as likely mechanisms driving this subgroup. That SPIB^low^/BATF^high^-ABC-DLBCL does not simply reflect a surrogate for STAT3 activation alone is indicated by the lack of enrichment of two signatures recently described as predictors of STAT3 activation in DLBCL as a whole (61). BATF has been previously identified as a target of the NFκB and STAT3 pathways in other cell systems (73–76), while IRF4 is seen as a principle target of NFκB activity in B-cell differentiation (77). In T-cells BATF and IRF4 cooperate with STAT3 acting potentially as pioneer factors (18). These transcription factors are thus likely to provide important hubs for signal integration both in post-germinal centre B-cell neoplasia and at the initiation of B-cell terminal differentiation.

From the point of view of clinical significance we have shown here that a high expression of *SPIB* and low expression *BATF* mRNA can identify a good prognostic group of DLBCL when treated with currently standard immunochemotherapy, R-CHOP. While this association could be observed in two of the existing data sets of R-CHOP treated DLBCL including the largest data set generated from fresh-frozen samples by the LLMPP (GSE10846) (29), and our population based cohort (GSE32918), it was not reproduced in the data set generated by The International DLBCL Rituximab-CHOP Consortium Program (GSE31312) (37). The latter includes the largest number of R-CHOP treated DLBCL cases analysed by gene expression profiling to date, and was generated on the same platform as the LLMPP data set, GSE10846, but derives from formalin-fixed paraffin embedded rather than fresh frozen samples. The reason why a good outcome group of ABC-DLBCL could not be identified from relative *SPIB* and *BATF* mRNA expression in GSE31312 could not be ascertained from the gene expression data, but it may reflect underlying differences in case selection. In this regard, it is notable that our data set GSE32918 is unique in representing the general population of DLBCL from a single geographically defined area (62), while other data sets derive from multi-institutional research consortia.

That SPIB^low^/BATF^high^-ABC-DLBCL is significantly associated with mutation of *MYD88* is consistent with the model recently proposed by Yang *et al.* of a role for SPIB/IRF4 heterodimers in repressing autocrine IFN secretion that limits ABC-DLBCL survival (16). In this regard, a striking feature of our results is the finding that in our population-based patient cohort SPIB^high^/BATF^low^-ABC-DLBCLs with a *MYD8*8-L265P mutation, detectable by Sanger sequencing, identifies a distinct group of ABC-DLBCL with a favourable outcome on current therapy. Indeed, the outcome of this small subgroup might be considered to represent ‘cure’. Since *MYD88-*L265P mutation status and mRNA expression levels are readily determined in a clinical setting it will be important to extend these observations in future, and evaluate whether this combination can be used to prospectively identify a subset of good risk ABC-DLBCL cases. However, we stress that given the small patient number in the retrospective analysis presented here, the result is at present only suggestive.

The significant association of *SPIB* expression with *MYD88* mutation status may also link to recent data demonstrating that MYD88-L265P remains dependent on TLRs in order to manifest its oncogenic potential (65). Further to this in a recent elegant study the impact of MYD88-L265P has been examined in murine models, demonstrating that the oncogenic potential of MYD88-L265P is also constrained by feedback mechanisms restricting NFκB pathway activation and by induction of apoptosis (66). SPIB is part of the core transcriptional network of plasmacytoid dendritic cells (48,78,79), which are particularly specialized for cellular responses following TLR ligation (80). Consistent with this extensive occupancy of SPIB in the promoters and immediate vicinity of *TLR4*, *TLR7* and *TLR9* as well as *MYD88* itself was evident from ChIP-seq data in OCI-LY3 and LY10 cells. While significant effects on *TLR* and *MYD88* mRNA expression were not observed on SPIB knockdown, this could be explained by the transient nature of SPIB knockdown in our experiments, and the potential for redundant regulatory input from other transcription factors. A particularly intriguing possibility is suggested by the preferential expression of *TCF4* (also known as *E2–2*) in the SPIB^high^/BATF^low^-ABC-DLBCL subgroup, and the presence of several SPIB binding peaks across the *TCF4* (*E2–2*) locus in OCI-LY3 and LY10 cells. Given the importance of E2–2 and SPIB in plasmacytoid dendritic cell development (48,79), these observations suggest that cooperation between these factors may contribute to the biology of the SPIB^high^/BATF^low^-ABC-DLBCL.

In conclusion, the data presented here define the relationship of IRF4 to its endogenous partners in ABC-DLBCL cell lines identifying BATF as a principle IRF4 partner in addition to SPIB in these models of lymphoma. Our data also indicate that a predominant input from SPIB correlates with a specific subgroup of primary ABC-DLBCL significantly associated with *MYD88* mutation and a better prognosis in the context of currently standard therapy. These data support a model in which overriding input from SPIB is not a unifying feature of ABC-DLBCL, but instead contributes to heterogeneity in this subset. Our findings identify disease heterogeneity in ABC-DLBCL intimately associated with the gene regulatory network controlling the initiation of plasma cell differentiation and the activated B-cell program.

## SUPPLEMENTARY DATA

Supplementary Data are available at NAR Online

SUPPORTING INFORMATION
