# Supplementary material for: SPIB and BATF provide alternate determinants of IRF4 occupancy in diffuse large B-cell lymphoma linked to disease heterogeneity
Source: Nucleic Acids Res. 2014 May 28;42(12):7591–610. doi: 10.1093/nar/gku451 (PMC4081075; doi:10.1093/nar/gku451)
Supplement: SUPPORTING INFORMATION [file supp_42_12_7591__index.html]

SPIB and BATF provide alternate determinants of IRF4 occupancy in diffuse large B-cell lymphoma linked to disease heterogeneity — SUPPORTING INFORMATION 

# SPIB and BATF provide alternate determinants of IRF4 occupancy in diffuse large B-cell lymphoma linked to disease heterogeneity

## SUPPORTING INFORMATION

**Files in this Data Supplement:**

- Supplemental Figures
- Supplemental Data
