# Supplementary material for: SPIB and BATF provide alternate determinants of IRF4 occupancy in diffuse large B-cell lymphoma linked to disease heterogeneity
Source: Nucleic Acids Res. 2014 May 28;42(12):7591–610. doi: 10.1093/nar/gku451 (PMC4081075; doi:10.1093/nar/gku451)
Supplement: SUPPORTING INFORMATION [file supp_gku451_nar-02714-x-2013-File012.pdf]

SPIB and BATF provide alternate determinants of IRF4 occupancy in Diffuse Large B-cell Lymphoma linked to disease heterogeneity.

Supplemental figures and methods

Supplemental figures pg 2

Supplemental methods pg 18

Supplemental Figure 1

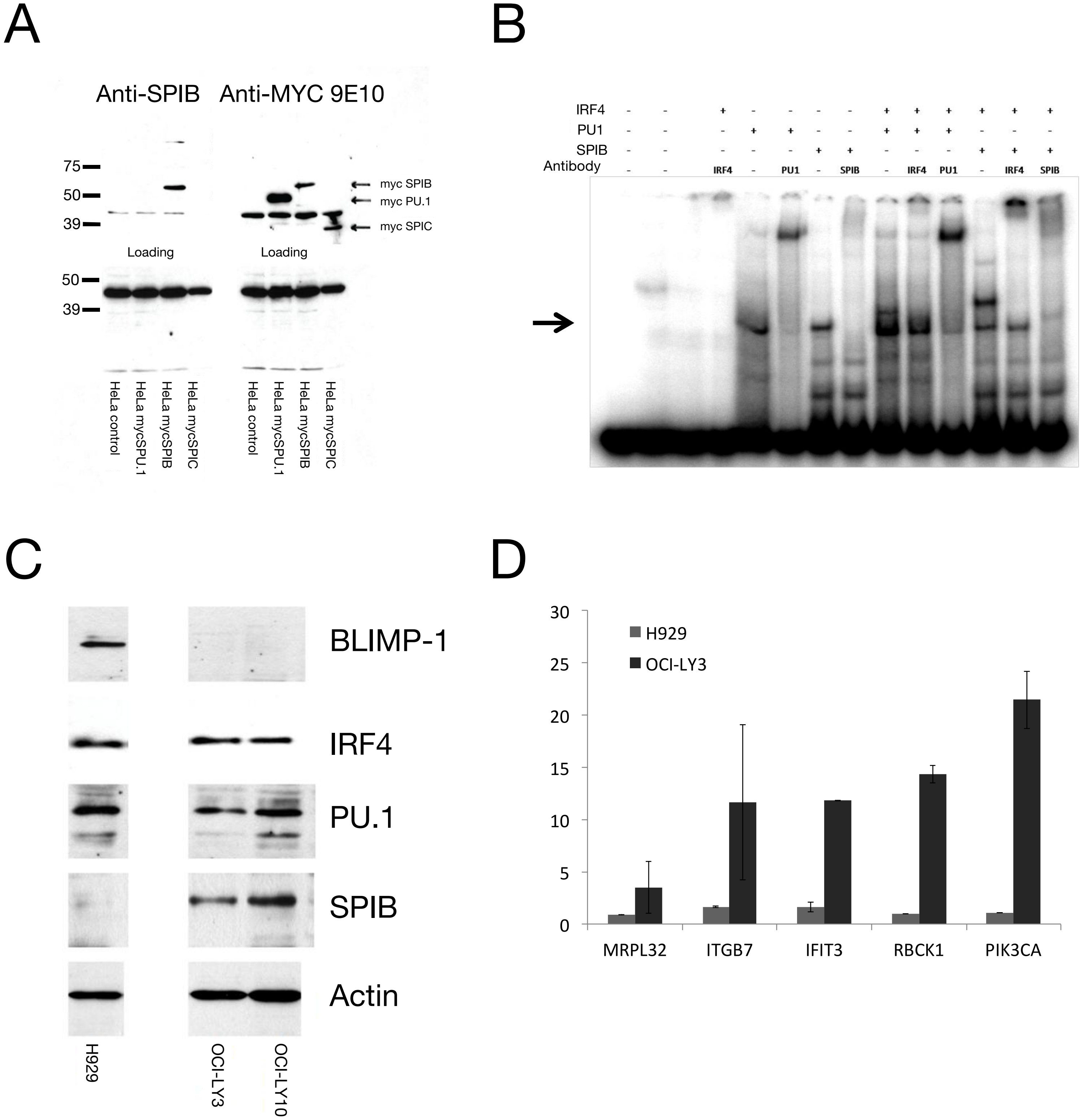

Supplemental Figure 1 (accompanies Figure 1). SPIB antibody validation (A) SPIB antisera was tested in Western blot against HeLa cells transfected with control expression vector or Myc-tagged expression vectors for PU.1, SPIB and SPIC. Left upper panel SPIB Western blot, right upper panel Myc-tag Western blot. Lower panels show loading control. (B) SPIB antibody was tested in EMSA using nuclear extract from COS cells transfected with expression vectors for IRF4, PU.1 and SPIB (indicated by +) and a probe encompassing the EICE site in the *PSMB8* promoter. Antibodies used for supershift are indicated beneath the composition of the extract. The arrow identifies the migration of the principle complex formed with PU.1 or SPIB alone. (C) Western blot of cell lines H929, OCI-LY3 and OCI-LY10 with the antibodies indicated on the right. (D) SPIB antisera was tested by ChIP in OCI-LY3 ABC-DLBCL cell line expressing high levels of SPIB, and H929 myeloma cell line expressing very little SPIB at selected potential target promoters as indicated. Average enrichment of specific antibody over control rabbit IgG are shown for duplicate ChIP samples, and are representative of at least three independent experiments.

Supplemental Figure 2

A

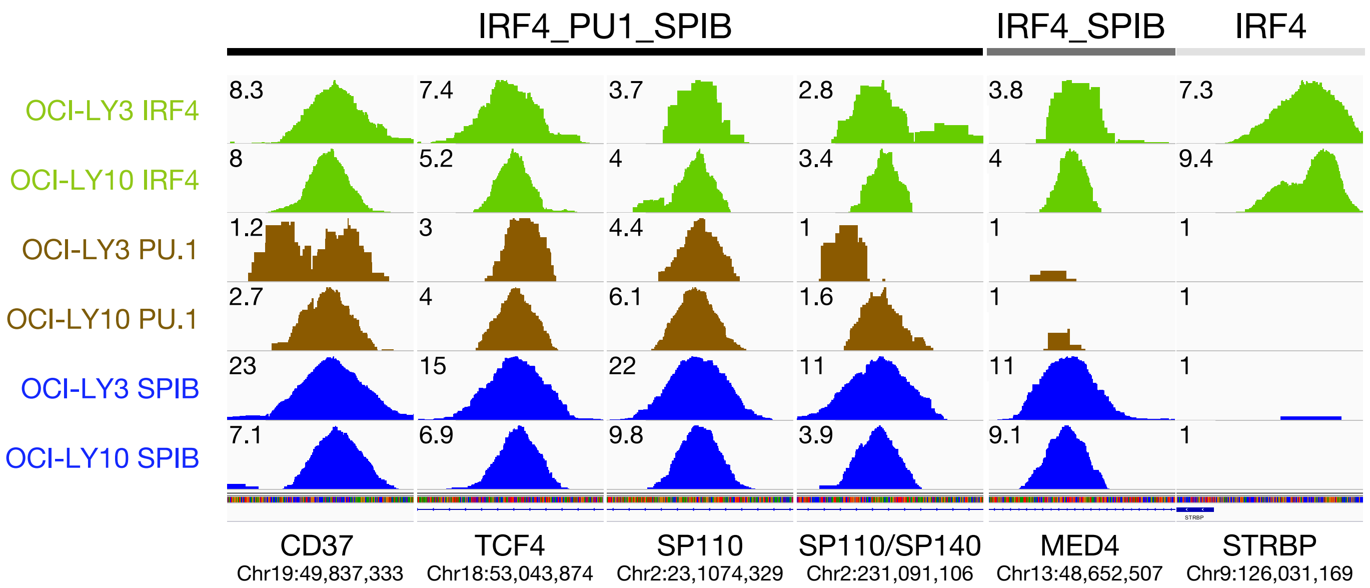

B

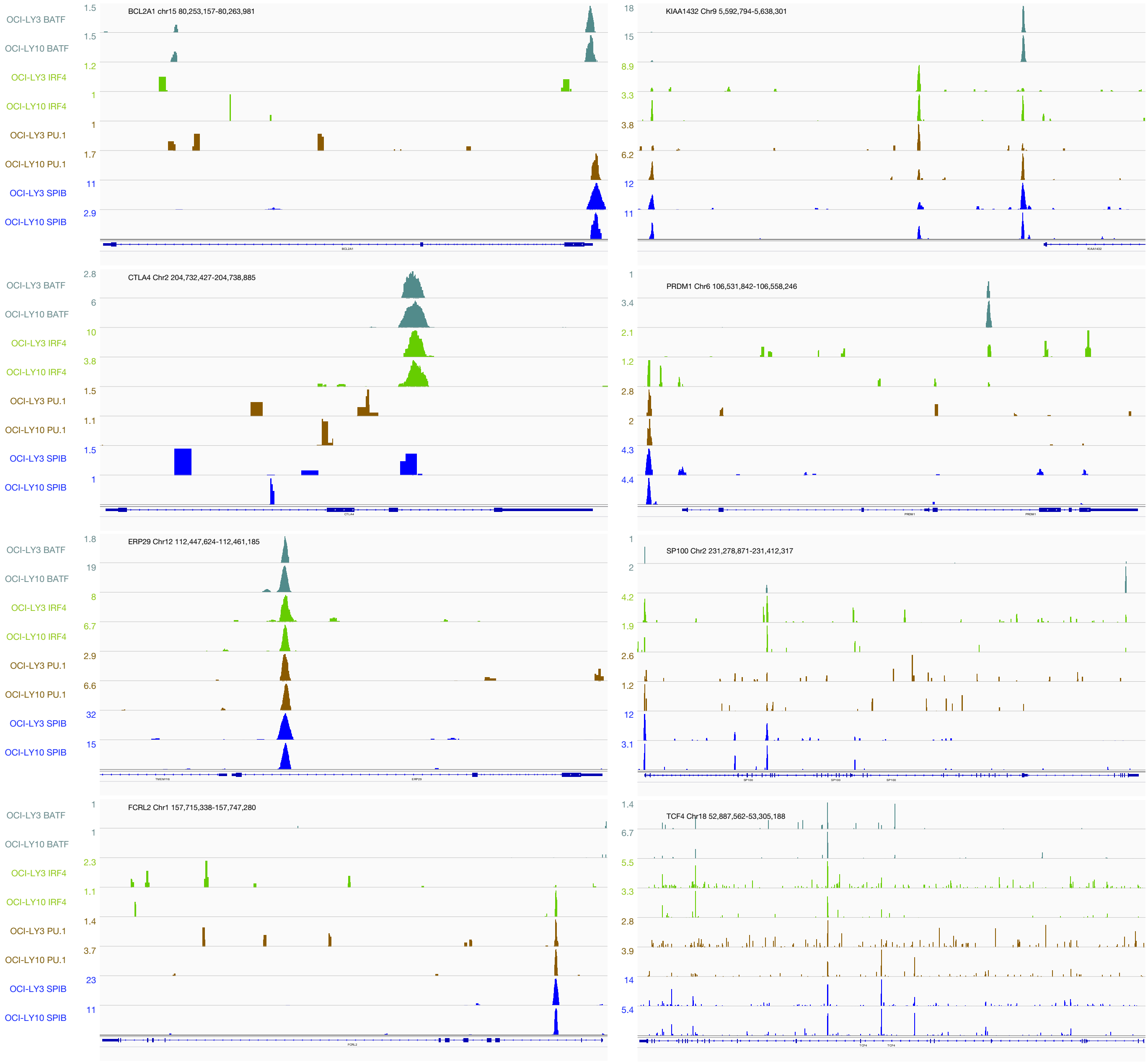

Supplemental Figure 2 (accompanies Figure 1). Further examples of SPIB genomic occupancy relative to PU.1 and IRF4 (A) Illustrates representative ChIP-seq results for SPIB, PU.1 and IRF4 from OCI-LY3 and LY10 cells at individual sites within the *CD37*, *TCF4* (*E2-2*), *SP110*, *SP140, MED4* and *STRBP* loci. The normalized read counts/million are shown in the top left of each track. (B) Illustrates ChIP-seq results spanning loci for the following genes: *BCL2A1, KIAA1432, CTLA4, PRDM1, ERP29, SP100, FCRL2* and *TCF4*.

Supplemental Figure 3

A

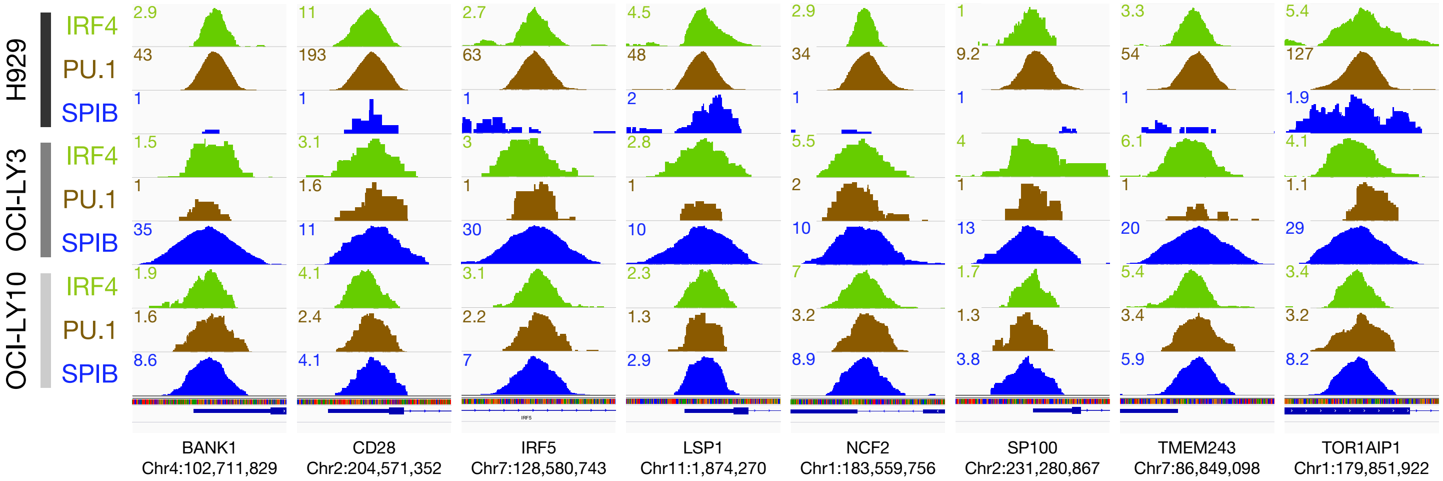

B

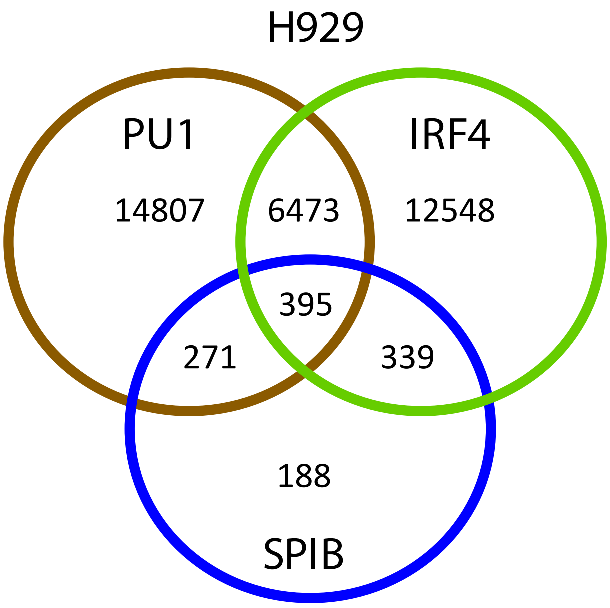

C

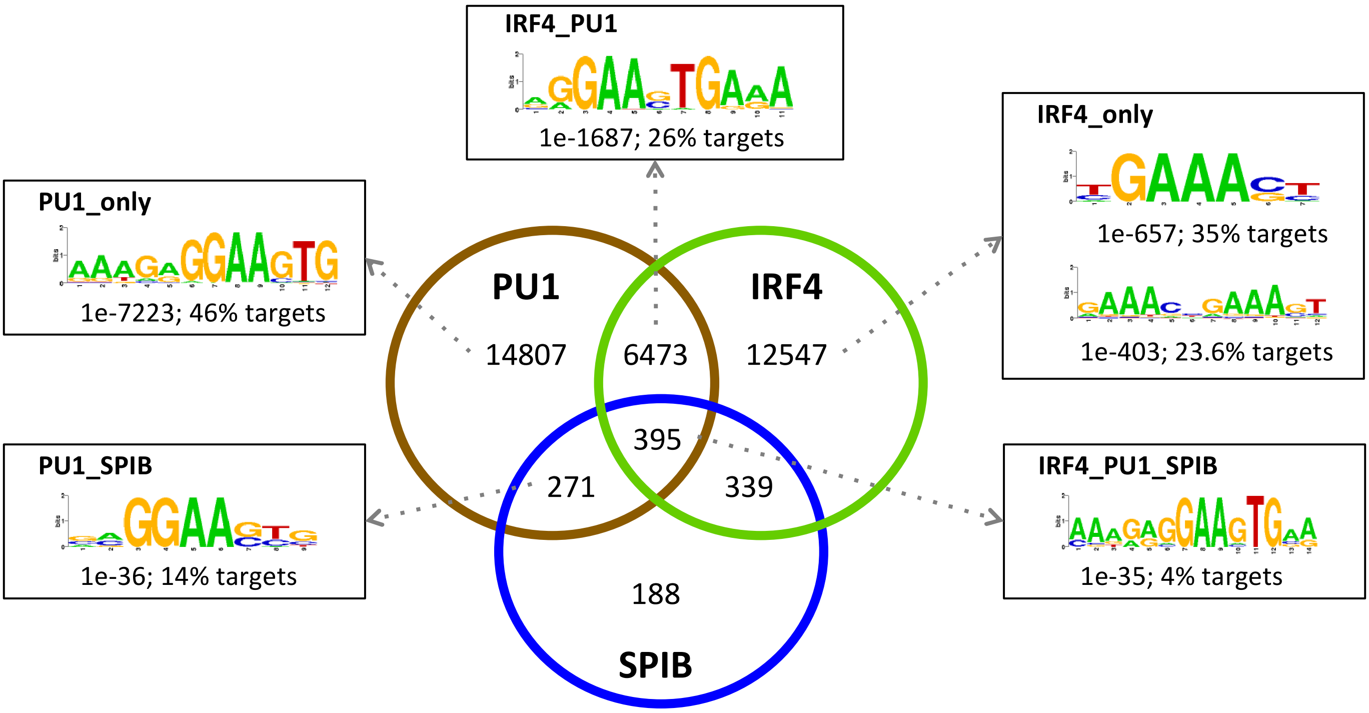

Supplemental Figure 3 (accompanies Figure 1 and 2). H929 ChIP-seq results (A) Illustrates representative ChIP-seq results for IRF4, PU.1 and SPIB in H929 cells. Occupancy at the same genomic location in OCI-LY3 and -LY10 is illustrated for comparison. Illustrates a switch in preferential ETS-factor occupancy from SPIB in ABC-DLCBL cell lines, to PU.1 in H929 myeloma cells. The normalized read counts/million are shown in the top left of each track allowing comparisons between cell types. (B) Venn diagram illustrates the overlap of IRF4, PU.1 and SPIB cistromes in H929 myeloma cells. (C) Illustrates principle motifs recovered by *de novo* motif discovery with HOMER, for different occupancy contexts in H929. The motif enrichment and percentage of peak regions with a motif match is illustrated.

Supplemental Figure 4

A

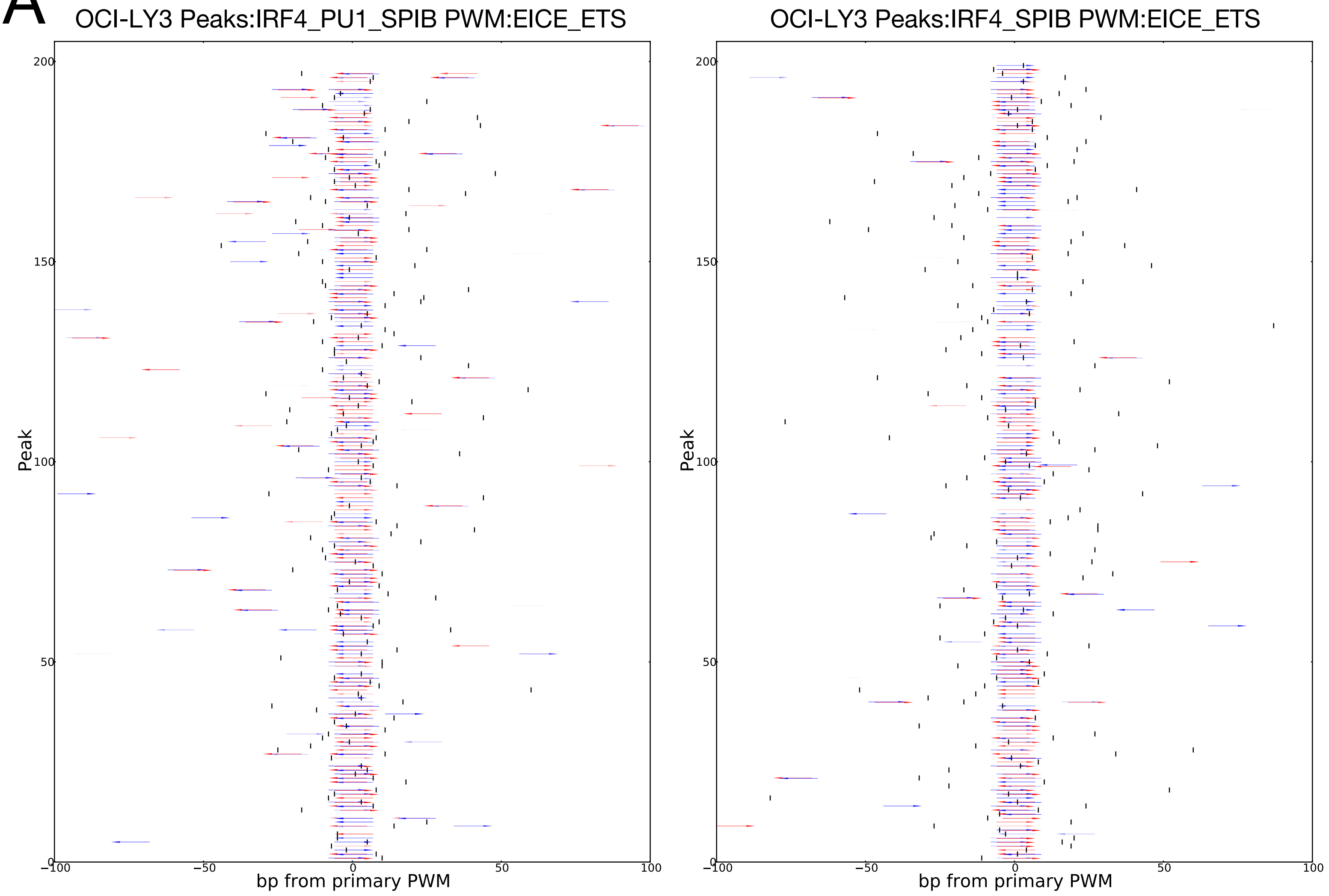

B

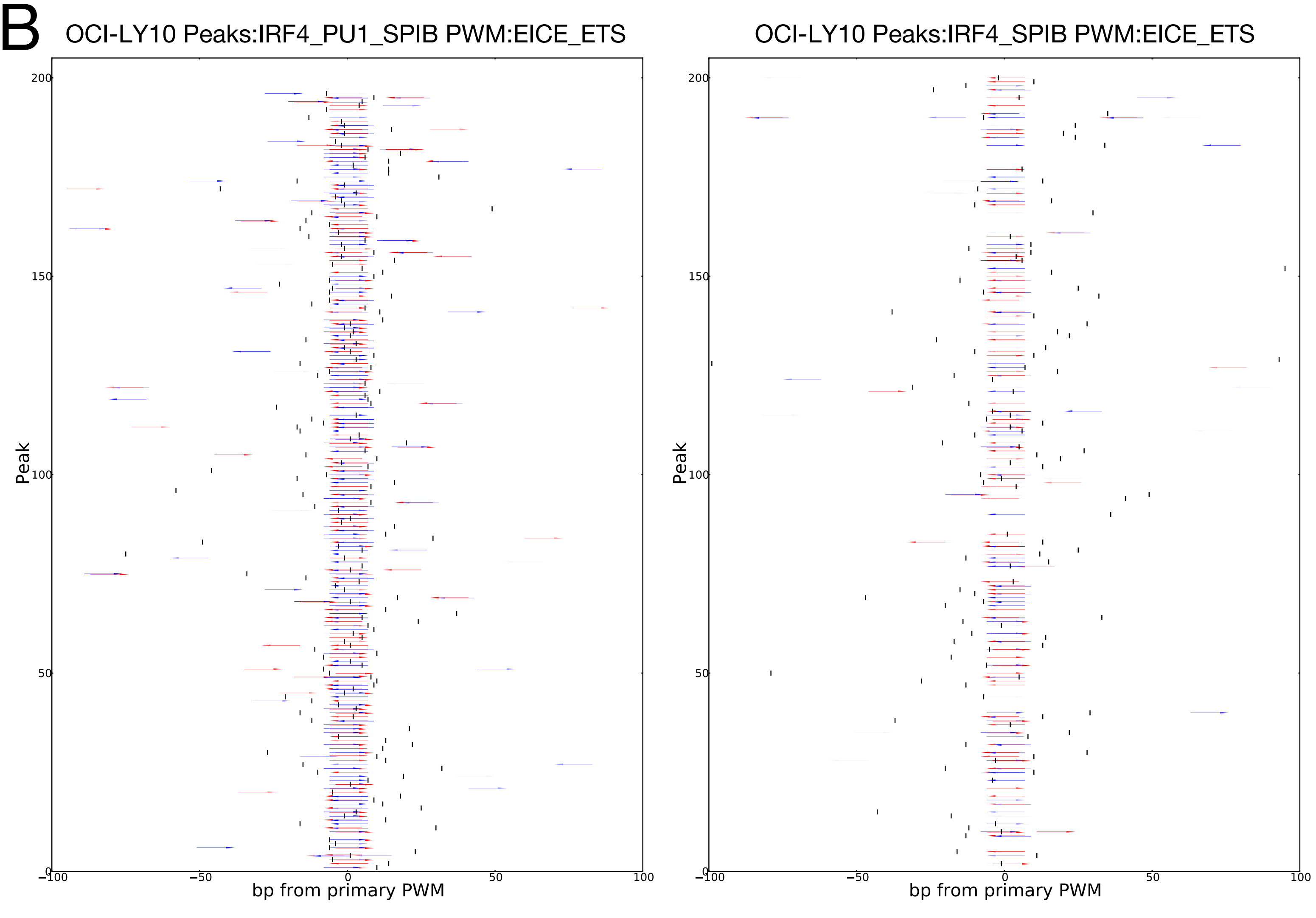

Supplemental Figure 4 (accompanies Figure 2). Motif usage in occupied peak regions. (A) and (B) show equivalent results for OCI-LY3 and OCI-LY10 data, respectively. Illustrated are motif matches to examples of EICE (red) and PU.1/SPIB motifs (blue) identified within the top 200 peak regions occupied by IRF4_SPIB_PU.1 (left) or IRF4_SPIB alone (right), the nearest motif match to the peak centre was identified as the primary motif, and secondary EICE and ETS motifs are illustrated in relative orientation and positioning within a 100 bp window. Intensity of arrow colour indicates quality of motif match, while arrowhead indicates relative motif orientation.

Supplemental Figure 5

A

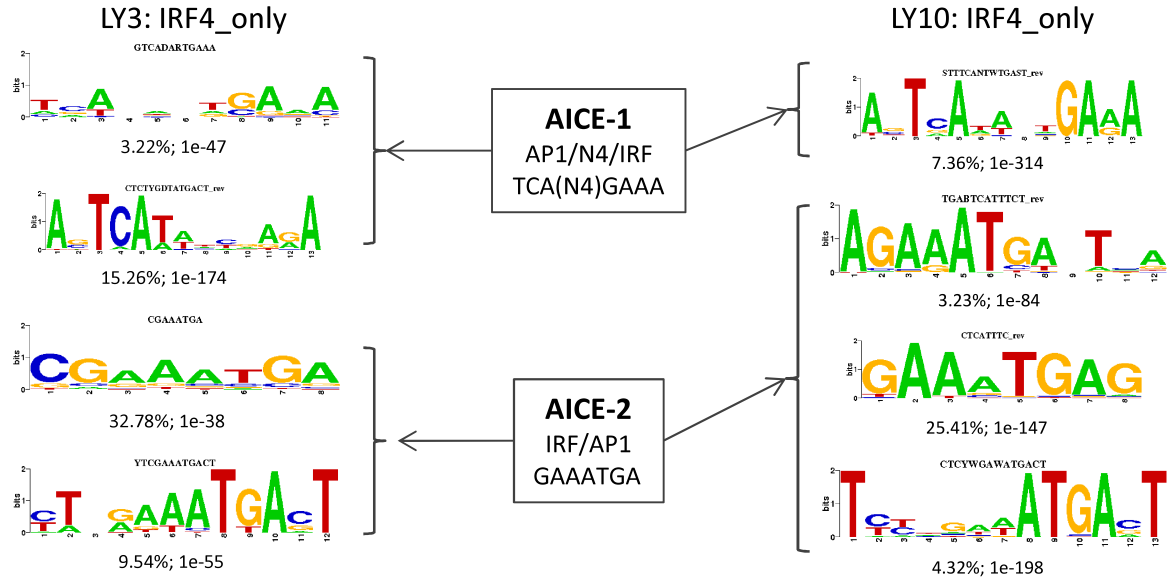

B

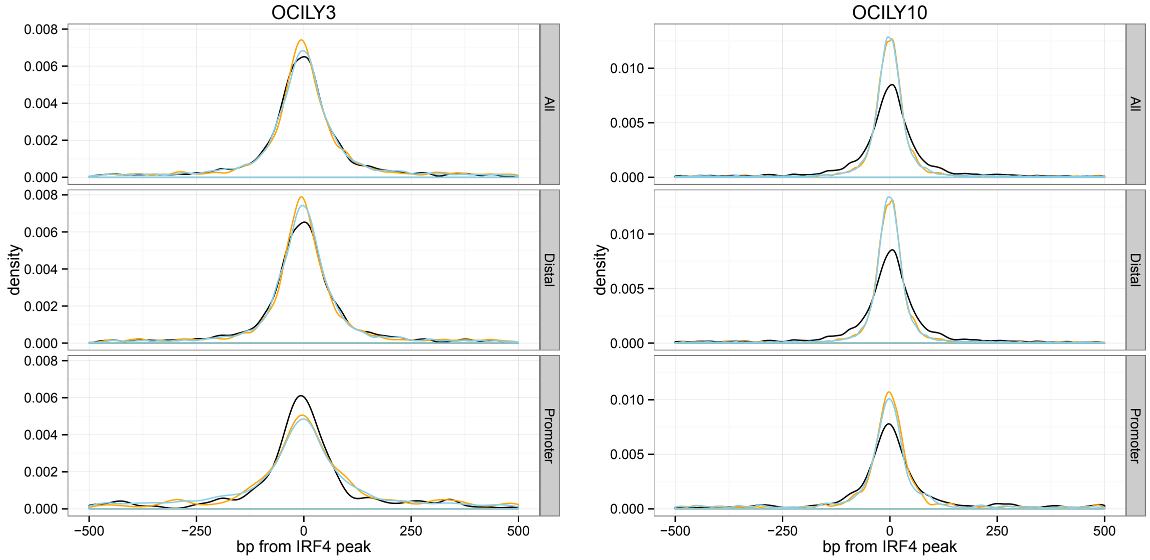

C

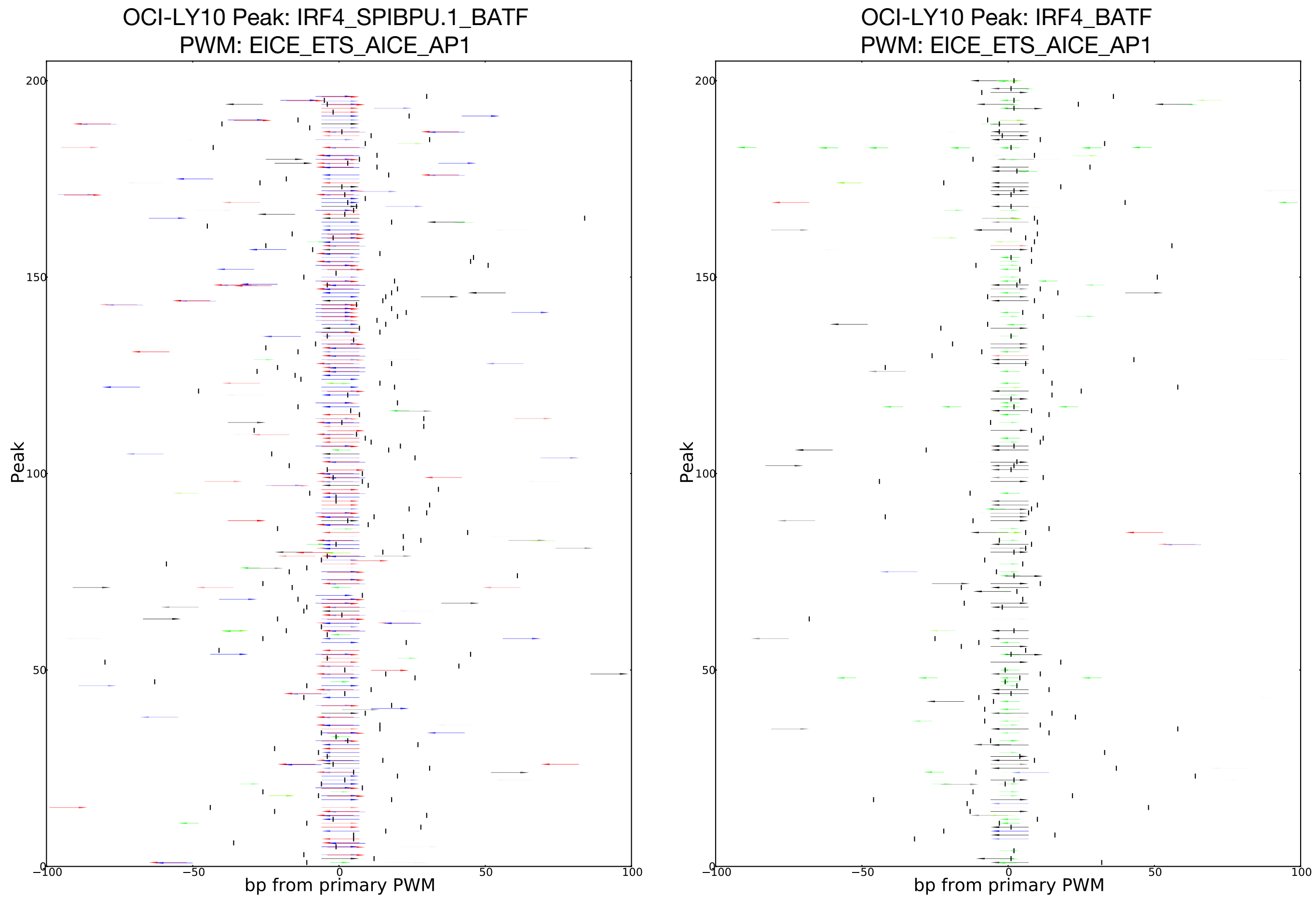

Supplemental Figure 5 (accompanies Figure 4 and 5). AICE motifs and BATF occupancy patterns in ABC-DLCBL cell lines.

(A) Representative motifs recovered from *de novo* motif detection using HOMER for each cell line (OCI-LY3 left; OCI-LY10 right) amongst IRF4_Only peaks (i.e. in the absence of either SPIB or PU.1 occupancy) illustrated are common recovered motifs corresponding to AICE-1 and AICE-2 motif variants. Motif enrichment and percentage of peak regions with motif match as determined by HOMER are indicated beneath each motif. Consensus sequences for AICE-1 and AICE-2 variants are shown for reference. (B) Density plots of the distribution of peak centres for BATF (black), SPIB (blue) and PU.1 (orange) relative to IRF4 peak centres. The x-axis shows 500bp up- and down-stream of IRF4 peak centres at 0. (C) Matches to examples of EICE (red), PU.1/SPIB (blue), AICE (black) and AP1 (green) motifs were identified within the top 200 peak regions occupied by IRF4, SPIB/PU.1 and BATF (left panel) or IRF4 and BATF alone (right panel), the nearest motif match to the peak centre was identified as the primary motif, and secondary motifs are illustrated in relative orientation and positioning within a 100 bp window. Intensity of arrow colour indicates quality of motif match, arrowhead direction indicates relative motif orientation and vertical lines indicate peak centres.

Supplemental Figure 6

A

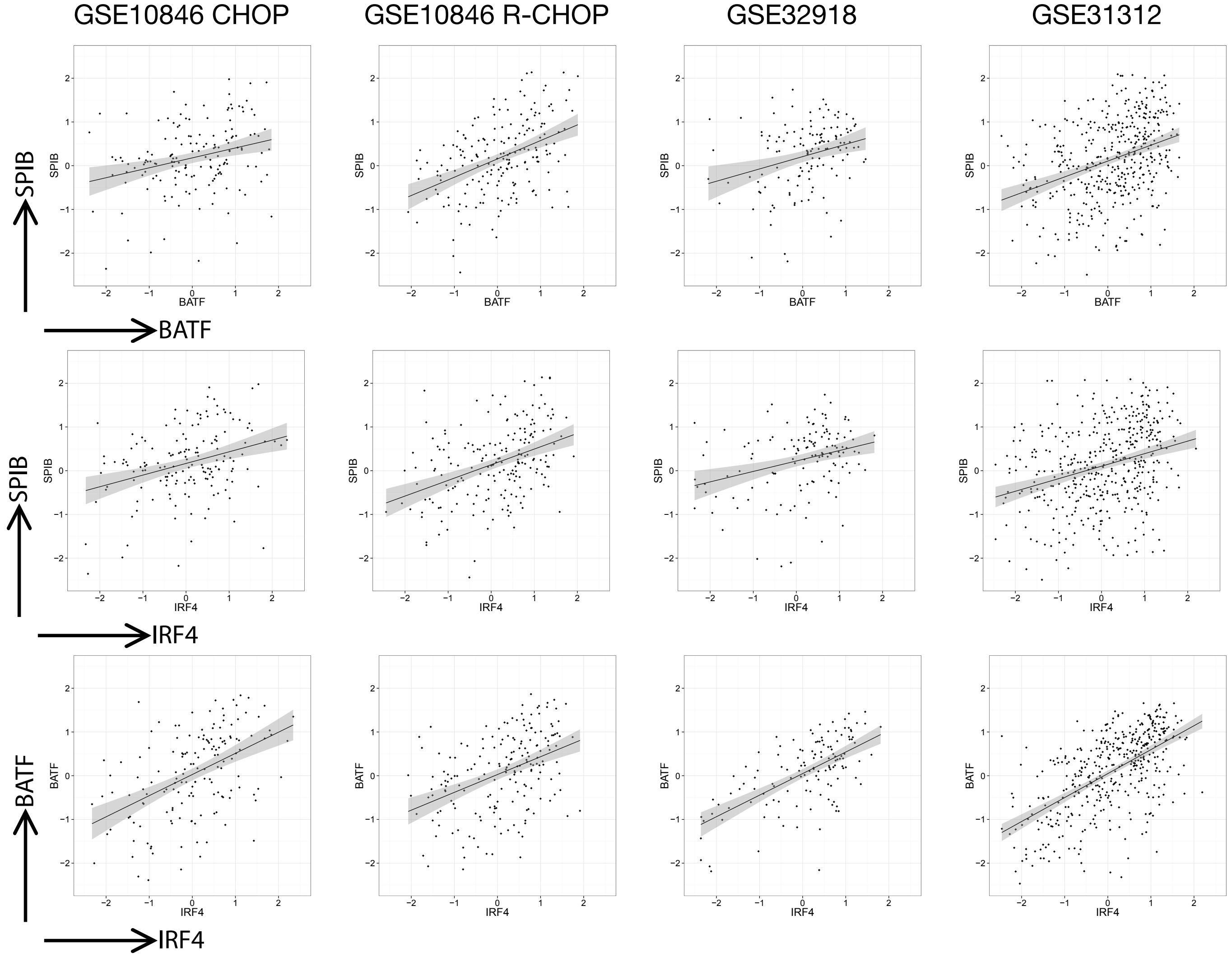

B

	Spearman's rank correlation		
**Data set**	**SPIB/BATF**	**SPIB/IRF4**	**BATF/IRF4**
Monti	0.50	0.52	0.50
GSE34171	0.48	0.41	0.45
GSE32918	0.31	0.33	0.56
GSE31312	0.38	0.32	0.59
GSE22895	0.32	0.47	0.63
GSE22470	0.28	0.43	0.42
GSE12195	0.54	0.57	0.67
GSE10846 CHOP	0.31	0.29	0.50
GSE10846 RCHOP	0.47	0.44	0.48
GSE4475	0.44	0.37	0.50
Avg	0.40	0.42	0.53
Median	0.41	0.42	0.50
StDev	0.094	0.090	0.080
Variance	0.009	0.008	0.006

Supplemental Figure 6 (accompanies Figure 8). Pairwise transcription factor correlations. (A) Panels illustrate the pairwise correlation of transcription factor expression in ABC-DLBCL cases of the GSE10846 CHOP, GSE10846 R-CHOP GSE32918 and GSE31312 datasets as indicated at the top of the figure. Upper panels - SPIB vs BATF; Middle panels – SPIB vs IRF4; Lower panels - BATF vs IRF4. Linear regression line is displayed with 95% confidence interval shown as grey area. (B) Summarises the Spearman correlations observed for the transcription factor expression values across all data sets examined.

Supplemental Figure 7

A

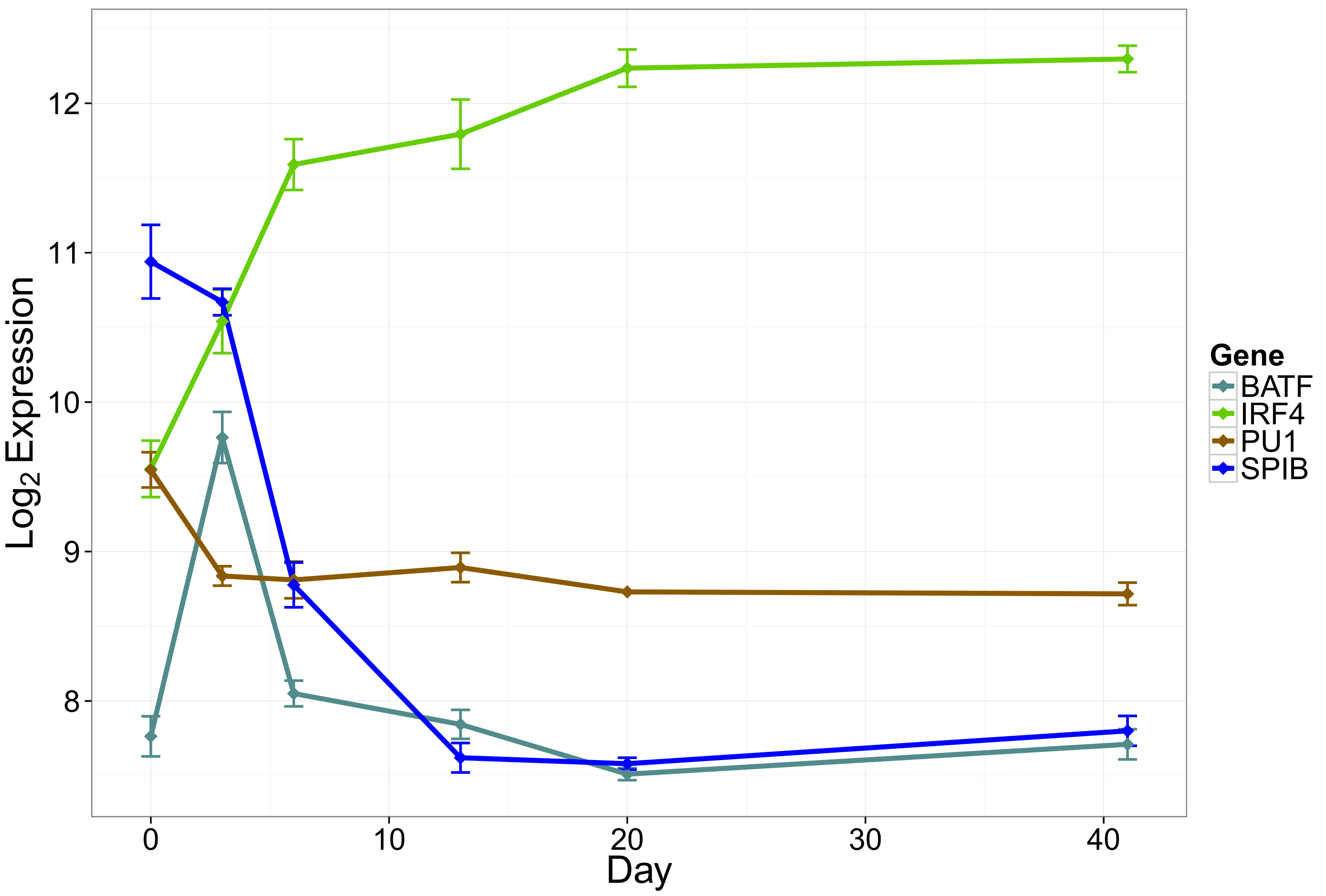

Supplemental Figure 7 (accompanies Figure 8). Pattern of transcription factor expression during B-cell to plasma cell differentiation. The expression pattern of *IRF4*, *SPIB*, *PU.1* and *BATF* in data set GSE41208 spanning human B-cell to plasma cell differentiation *in vitro* is illustrated; IRF4 - green line; SPIB - blue line; PU.1 - brown line; BATF - teal line. Error bars show SEM.

Supplemental Figure 8

A

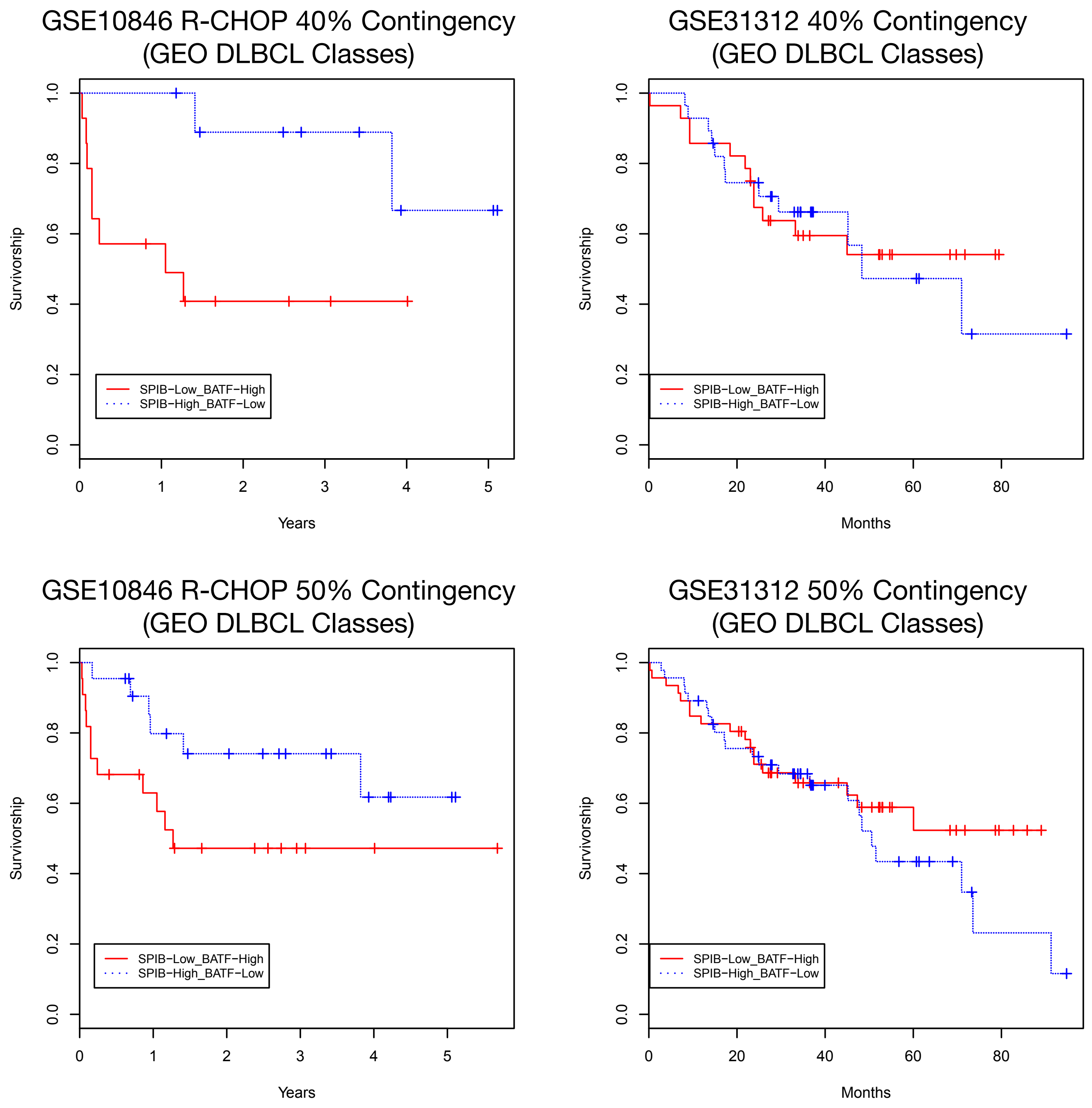

B

Dataset	Contingency %	2-Way Contingency Table<br>SPIB-High BATF-Low<br>Hazard Ratio	95% Conf Intervals		*P*-Val	logrank
GSE32918	40	0.20	0.04	1.05	0.06	0.04
GSE32918	50	0.29	0.09	0.89	0.03	0.02
GSE10846	40	0.19	0.04	0.95	0.04	0.03
GSE10846	50	0.30	0.10	0.91	0.03	0.02
GSE10846 GEO DLBCL Class	40	0.19	0.04	0.91	0.04	0.02
GSE10846 GEO DLBCL Class	50	0.40	0.15	1.10	0.07	0.07
GSE31312	40	1.26	0.55	2.92	0.58	0.58
GSE31312	50	1.81	0.90	3.64	0.10	0.09
GSE31312 GEO DLBCL Class	40	1.07	0.48	2.38	0.87	0.87
GSE31312 GEO DLBCL Class	50	1.29	0.69	2.40	0.43	0.43

GSE32918 MyD88 Mutations	WT<br>Hazard Ratio	95% Conf Intervals		*P*-Val	SPIB-High L265P<br>Hazard Ratio	95% Conf Intervals		*P*-Val	logrank
L265P Vs WT	1.52	0.73	3.16	0.26	-	-	-	-	0.26
SPIB-High_L265P vs L265P vs WT	0.79	0.37	1.68	0.54	0.08	0.01	0.62	0.02	0.01

Supplemental Figure 8 (accompanies Figure 9). Additional outcome data for ABC-DLBCL divided by SPIB and BATF expression for GSE10846, and GSE31312. (A) Shows Kaplan-Meier survival analysis for SPIB^{high}/BATF^{low} and SPIB^{low}/BATF^{high} subgroups of GSE10846 R-CHOP component using ABC-DLBCL class-assignments in GEO rather than those assigned by our classifier tool DAC. The results for using either top and bottom 50% or top and bottom 40% of *SPIB* and *BATF* expression to identify subgroups are shown on the left. Both separations follow the same trend but with a greater significance when using the more stringent threshold. In the right hand two panels the Kaplan-Meier survival analysis for GSE31312 is shown, using ABC-DLBCL class-assignments in GEO, as discussed in the text in this data set no significant difference in survival between ABC-DLBCL cases separated by *SPIB* and *BATF* expression could be observed. (B) The upper panel summarizes the hazard ratios, confidence intervals, p- and log rank values observed for the two-way comparison of SPIB^{high}/BATF^{low} and SPIB^{low}/BATF^{high} ABC-DLBCL subgroups. The lower panel shows hazard ratios, confidence intervals, p- and log rank values observed for the two-way comparison with the ABC-DLBCL class of *MYD88*-L265P mutation status vs *MYD88* wild type (*MYD88* mutations other than L265P included in the wild type category to reflect potential application of targeted mutation detection in a clinical setting) or the three-way comparison with ABC-DLBCL class of *MYD88*-L265P-SPIB^{high}/BATF^{low} vs all other *MYD88*-L265P vs *MYD88* wild type (again including all *MYD88* mutations other than L265P in the wild type category).

Supplemental Methods

Primers used for cloning:

SPIB.EcoR1.F-TTAAGAATTCGCCACCATGCTCGCCCTGGAGGCT;

SPIB+51.BglII.R- TTAAGAATTCGCCACCATGCTCGCCCTGGAGGCT;

SPIB.BglII.R-ATATAGATCTTCAGGCCCGGCGGACTG;

SPI1.EcoRI.F-TTAAGAATTCGCCACCATGGAAGGGTTTCCCCTC;

SPI1.BamHI.R-ATATGGATCCTCAGTGGGGCGGGTGG;

IRF4.EcoR.F-TTAAGAATTCGCCACCATGAACCTGGAGGGCGGC;

IRF4.BamH1.R-ATATGGATCCTCATTCTTGAATAGAGGA

Probes used in EMSA:

SETBP1.WT.F-AAAGGATGACTCAAAATGAAAGTTTCAAAG;

SETBP1.WT.R-CTTTGAAACTTTCATTTTGAGTCATCCTTT;

SETBP1.MT.F-AAAGGATGACTTTAAATGAAAGTTTCAAAG; S

ETBP1.MT.R-CTTTGAAACTTTCATTTAAAGTCATCCTTT;

FOXO3A.WT.F-ACCACACTACTTTCATTCTGAGTAATGTAA;

FOXO3A.R.F-TTACATTACTCAGAATGAAAGTAGTGTGGT;

FOXO3A.MT.F-ACCACACTACTTTCATGCGGTGTAATGTAA;

FOXO3A.MT.R-TTACATTACACCGCATGAAAGTAGTGTGGT

Primers for ChIP

MRPL32_F1	TCTTTACCCGAAGAGCCAAA
MRPL32_R1	GCTCTTGGAGCAGCTCTGAC
ITGB7_F-1804	GCCAGGACCTATACCCTTCC
ITGB7_R-1999	TCCTGGCTCTCTTCATTGGT
IFIT3_F2	TGATTCCAAGCAAATGCTGA
IFIT3_R2	AACATGTTCCTGGCGTGAGT
RBCK1_F1	GATGAGGCGACAATTTTTCC
RBCK1_R1	GTTTCAAGGCAAAGCCCTCT
PIK3CA_F-497	CAGGCGCAGTAGCACATATT
PIK3CA_R-381	GAGGGGCAGAGCCTACAATC

Chr10:124134872 F	TGGTTTGTGGTTTGTTTCAGA
Chr10:124134872 R	TTCTACCAAAGGCGGTAAACA
Chr18:12309614 F	GTCCTCACCGGGAACAAGT
Chr18:12309614 R	CCATGTGCAGTCTGAGAACC
Chr11:4720870 F	CATGCTTATTCCTTGCACATAAA
Chr11:4720870 R	AGTCACAGCCCTACAATTTGC
Chr18:42289582 F	AAACCGGGATTACTGCTGAG
Chr18:42289582 R	TGCAACGATGAGGAAGAGG
FAM129C F	GCAGGAGATGGGAATTGAAA
FAM129C R	CTTCCGCTCAGCTCTCTTTG
CD23 F	TCTCTTCCTGCTTAAACCTCTGTCT
CD23 R	TTAGTGGAGTTTGGAGCCTGTGT
TLR4 -178 F	CCAAGCCCAGGCAGAGGT
TLR4 -88 R	AAACGCCTGCAGACCAGTG
Chr5 141303203 F	ACTTCCTCTCTGCAGCGC
Chr5 141303203 R	GTACCCGAGGAAGCTCTGAG
Chr1 110090938 F	TCGCCGGAAGTGTCGTAAAC
Chr1 110090938 R	CCTCTCCGTCAGCTCCCTTA
Chr1 156025578 F	ACTCACAGAACCATGGCAGT
Chr1 156025578 R	AGTTCACAATCTAATGGTGACTTCC

Primers and PCR conditions used for amplification of *MYD88* coding exons

Gene	Exon	Primer[1]	Sequence (5'-3')	Amplicon size (bp)	PCR annealing temperature (°C)	PCR condition
MYD88	E1-1	Forward	CTCGGGGCTCCAGATTGTA	327	58	PCR was carried out in a 10μl reaction mixture with 5-10ng template DNA and AmpliTaq Gold 360 (Applied Biosystem) master mix plus GC-enhancer according to the manufacturer's instructions. The PCR conditions were 95°C for 10 min to activate the enzyme, followed by 40 cycles of denaturation at 95°C for 20 sec, annealing at 58-62°C (depending on the primer set) for 20 sec, and extension at 72°C for 45 sec.
		Reverse	GCCGGATCTCCAAGTACTCA			
	E1-2	Forward	GCTGCTCTCAACATGCGAGT	317	62	
		Reverse	GGAAAGTCAGCCTCCTCACC			
	E2	Forward	CTGGATCCTGACTGTGGGTAA	281	62	
		Reverse	GCTTCAAACACCCATGCTCT			
	E3	Forward	TCTGACCACCACCCTTGTG	264	62	
		Reverse	CAGGGCAGGGCTTCATGC			
	E4	Forward	GGCCCTTCCTGAAGCTATTC	270	62	
		Reverse	TGGTACTGCATCCACAGTCC			
	E5	Forward	GTTGAAGACTGGGCTTGTCC	292	59	
		Reverse	AGGAGGCAGGGCAGAAGTA			

Bioinformatic methods

ChIP-Seq

Overview of ChIP-seq data:

ChIP	TotalReads	TotalAligned	%Aligned	TotalPos	%Pos	TotalNeg	%Neg
H929 Input	31,547,003	24,679,253	78.23	12,337,167	49.99	12,342,086	50.01
H929 IRF4	32,217,321	27,308,519	84.76	13,649,050	49.98	13,659,469	50.02
H929 PU1	29,392,695	24,407,405	83.04	12,196,016	49.97	12,211,389	50.03
H929 SPIB	29,391,578	23,739,271	80.77	11,860,342	49.96	11,878,929	50.04
OCILY3 Input	35,482,440	30,927,579	87.16	15,471,925	50.03	15,455,654	49.97
OCILY3 IRF4	34,319,987	26,126,842	76.13	13,031,771	49.88	13,095,071	50.12
OCILY3 PU1	29,930,730	20,732,913	69.27	10,363,621	49.99	10,369,292	50.01
OCILY3 SPIB	35,889,335	26,714,556	74.44	13,372,706	50.06	13,341,850	49.94
OCILY10 Input	33,739,393	28,358,599	84.05	14,183,740	50.02	14,174,859	49.98
OCILY10 IRF4	30,282,459	19,424,800	64.15	9,706,534	49.97	9,718,266	50.03
OCILY10 PU1	36,839,678	21,382,085	58.04	10,692,488	50.01	10,689,597	49.99
OCILY10 SPIB	36,969,891	23,794,577	64.36	11,893,987	49.99	11,900,590	50.01
OCILY3 BATF	13,490,958	11,054,333	81.94	5,525,932	49.99	5,528,401	50.01
OCILY10 BATF	21,263,180	17,750,229	83.48	8,872,347	49.98	8,877,882	50.02
OCILY3 Input Scrm	23,765,589	18,625,116	78.37	9,310,239	49.99	9,314,877	50.01
OCILY3 Input SPIBkd	27,615,812	22,438,885	81.25	11,219,476	50.00	11,219,409	50.00
OCILY3 IRF4 Scrm	21,523,644	18,295,567	85.00	9,144,700	49.98	9,150,867	50.02
OCILY3 IRF4 SPIBkd	27,547,175	22,986,237	83.44	11,490,593	49.99	11,495,644	50.01
OCILY3 PU1 Scrm	24,454,075	20,671,957	84.53	10,335,137	50.00	10,336,820	50.00
OCILY3 PU1 SPIBkd	33,759,309	28,669,665	84.92	14,332,716	49.99	14,336,949	50.01
OCILY3 SPIB Scrm	24,959,661	21,544,244	86.32	10,772,432	50.00	10,771,812	50.00
OCILY3 SPIB SPIBkd	30,640,580	25,994,396	84.84	12,997,014	50.00	12,997,382	50.00

ChIP-Seq alignment and peak discovery

Before aligning, reads were trimmed to remove adapters and low confidence regions using a python script. A 4 base sliding window was run along each read, if the average Q Phred score for a window was < 20 the read was trimmed at the window start, finally any match to adapter sequences were trimmed.

Trimmed reads were aligned using Bowtie2 with the --very-sensitive parameter (1). The resultant SAM files were converted to BAM using Samtools with the quality filter set to 20 (i.e. -q 20) (2). The BAM files were analysed for peaks using GEM, with quality filter set to 1 (i.e. -q 1) (3).

The resultant BAM files were converted to BED files and read cross-correlation was assessed using MaSC (4). Reads were extended to the estimated fragment length, and a scaled (reads per million; rpm) BED file generated. This was converted to a coverage file using the UCSC genomeCoverageBed tool and then to a BigWig file using UCSC bedGraphToBigWig (5). These

coverage files were used for visualisation via IGV and the extended BED files for fold change analysis (see Fold-change analysis) (6).

ChIP-seq peak overlap

The output from GEM was analysed using a python script to find overlaps between the different transcription factors (TFs). The peak centres for all TFs were ordered per chromosome. Starting at the beginning of each chromosome peaks were added to a cluster. New peaks were only added to the cluster if the distance between them and the first peak was < 250 bp, else a new cluster was started. As new clusters were generated if peaks in the earlier cluster were closer to the new cluster's centre they were moved into the new cluster (thus a peak can only belong to one cluster). The minimum GEM Q score for a peak to start a new cluster was set to >= 2, however, this was lowered to 1 for the addition of peaks to existing clusters.

Peak annotation

Version 14 of the Gencode gene annotation data was downloaded from UCSC, the genes were re-annotated using the HUGO Gene Nomenclature Committee annotations (2013/06/03 version) (5, 7). Each GEM peak was assigned the nearest gene as its primary gene, peaks lying 2000 bp up/down-stream of a TSS were deemed to be in promoters, those outside this region but within a gene body were termed intragenic and all others were termed intergenic.

Motif detection and enrichment analysis

BED files were generated for GEM peaks, and peak overlap sets, -/+ 125 bases around each peak centre (or overlap cluster centre). These were analysed for *de novo* motifs of length 8 – 14 using HOMER (8).

PWM analysis

De novo position weight matrices (PWM) from HOMER were scanned across the entire genome using MOODS(9). For each PWM the matches that accounted for <= 30% of total genome wide matches were retained.

Plots of PWM occurrence around ChIP-seq peak centres were generated using a python script. Using the results from MOODS the top 200 most significant peaks for each peak set (see ChIP-seq peak overlap) were searched for PWM matches -/+ 200 bp around each peak centre. The nearest PWM to the peak centre was assigned as the primary match. All matches within -/+ 100 bp around the primary motif were retained.

The resulting matches were drawn using Matplotlib, with the primary match in the centre, significance indicated by intensity of colour and orientation by arrow direction(10). The peak centres are represented as short vertical lines.

ENCODE analysis

In addition to locally generated data, BED files were downloaded for BATF, IRF4 and PU.1 from the ENCODE data set GM12878 (11). The genome was split into 100 bp windows, and any window overlapped by a TF's BED file was assigned a 1, while those without were set to 0. This 2D matrix of TF's vs bp windows was then used to calculate a Pearson's correlation using numpy.

Fold-change analysis

For the SPIB knock-down ChIP-seq data sets we wanted to explore the quantitative occupancy differences for IRF4, PU.1 and SPIB upon SPIB knockdown (e.g. IRF4_Scrm vs IRF4_SPIBkd). This was accomplished with a simple python script. Peaks generated by GEM and the extended reads in the form of BED files (see ChIP-Seq alignment and peak discovery) were used as input.

For a given transcription factor the peaks from the scramble and the SPIB_knock-down ChIP-seq were merged to form the union, i.e. all peak locations before/after knockdown in one file. Then for each of peak locations the sum of reads (as reads per million; rpm) was calculated -/+ 50bp around the peak centre in the scramble and SPIBkd data set, the input read count in the same region subtracted from each and a pseudocount added (0.25). Finally, the log fold-change was calculated between the scramble/SPIBkd conditions.

Several filtering steps were employed to minimise artefacts. Only peaks with a GEM log p-value of >= 2 were used. Peaks for which the read count in either scramble/SPIBkd < 1 rpm (after input subtraction) were discarded.

In certain contexts we wanted to be able to study the quantitative changes to a single TF in isolation, e.g. IRF4_Only. Here our main aim was to be confident that this TF was binding alone (i.e. for IRF4_Only that PU.1 and SPIB were not binding), this could be confounded by GEM not calling peaks for the other TFs or calling peaks with a log p-value < 2. Thus for such cases the read numbers for the TFs that were to be excluded were also calculated (e.g. for IRF4_Only read numbers for SPIB_ Scrm, SPIB_SPIBkd, PU1_Scrm and PU1_SPIBkd were also calculated) and if the maximum read number > 0.5 rpm the peak was excluded.

Gene expression data

RNA was run on Illumina HumanHT-12 v4 expression bead arrays, scanned with the Illumina BeadScanner and initial data processing carried out with Illumina GenomeStudio using the Gene Expression Module. The resulting final-report file was re-annotated using the NCBI API and then using the HUGO Gene Nomenclature Committee annotations (2013/06/03 version).

Differential gene analysis

The re-annotated data was quantile normalised using the Lumi package for R(12). A linear model was fitted to the gene expression data using the R Limma package(13). Differentially expressed genes between scramble and SPIB knock down were gauged using the Limma empirical Bayes statistics module, adjusting for multiple testing using Benjamini & Hochberg correction.

DLBCL data sets and analysis

The diffuse large b-cell data sets used are described in full in our previous work(14).

Contingency table groups

The ABC-DLBCL data was ranked by SPIB and BATF, the top/bottom 50% of each assigned to high/low and then split into 4 groups (high/high, high/low, low/high and low/low), e.g. $SPIB^{high}/BATF^{low}$.

Survival analysis

For each DLBCL data set the survival of the $SPIB^{high}/BATF^{low}$ and $SPIB^{low}/BATF^{high}$ groups were compared. The Survival library for R was used to analyse this right-censored survival data, overall survival was estimated using the Kaplan-Meier method, modelled with Cox Proportional Hazards technique (15).

Meta-profile generation

The 10 DLBCL data sets were previously classified into ABC/GCB/TypeIII (14). For each data set the ABC classified patients were split into contingency groups (see Contingency table group). The $SPIB^{high}/BATF^{low}$ and $SPIB^{low}/BATF^{high}$ groups were used for differential gene expression analysis (see Differential gene analysis). The genes up-regulated (p-value < 0.05) in $SPIB^{high}/BATF^{low}$ and $SPIB^{low}/BATF^{high}$ were used to create a meta-profile by finding genes commonly differentially expressed.

Comparison of meta-profile against B-cell differentiation

Meta-profile genes up-regulated in >= 4 data sets were compared against the top 15% most variable probes (n=3200) from a B-cell differentiation time series (GSE41208) (16). The probes were merged per gene by taking the median expression (n=2743). The significance of overlap was then assessed using a hypergeometric distribution.

To assess potential skewing of meta-profile genes to stages of B-cell differentiation the 2743 genes from the differentiation time course were split into groups according to their maximal expression: Day0 → BC, Day3 → ABC, Day6 → PB and >Day6 → PC. The number of meta-profile genes up-regulated in >=4 data sets that fell into each group was compared against $1x10^7$ random selections of genes (chosen from the 2743, n=size of meta-profile) and p-values generated.

Comparison of meta-profile against ChIP-seq data

Meta-profile genes up-regulated in >= 4 data sets were compared against genes occupied by BATF and SPIB.

Genes with significant peaks (GEM log p-value >=2) lying -5kb upstream of TSS or intragenic were deemed to be occupied by that TF. All genes with a log_2 expression >= 7 were considered as the total gene pool. The significance of overlap between the meta-profile genes and the ChIP-seq occupied promoters was then assessed using a hypergeometric distribution.

Enrichment of gene sets

A data set of 14,116 gene signatures was created by merging signatures downloaded from http://lymphochip.nih.gov/signaturedb/(**SignatureDB**),http://www.broadinstitute.org/gsea/msigdb/index.jsp MSigDB V4 (**MSigDB C1--C7**), http://compbio.dfci.harvard.edu/genesigdb/ Gene Signature Datbase V4 (**GeneSigDB**) and 4 individual papers (17–23).

Gene signature enrichment was carried out for the $SPIB^{high}/BATF^{low}$ and $SPIB^{low}/BATF^{high}$ meta-profiles (see Contingency table groups) using a hypergeometric analysis. To avoid any bias BATF/SPIB were removed from the gene signatures.

Figure generation

Figures 1C, 3A, 7C, 8A, 8C and Supplemental Figures 5B, 6A, 7A were generated using ggplot2 (24). Wordles in Figures 3B and 8D were generated using http://www.wordle.net/. The heatmap in Figure 7A was created using GenePattern with text highlighted in Adobe Illustrator(25). The heatmap in Figure 8D was created using a customised version of ggplot2 heatmap.2. ChIP-seq visualisations were generated using output from IGV, with the floor set to 0.25 rpm and the ceiling rpm displayed (6).

Motif logos throughout the figures were created using STAMP (26).

References

1. Langmead, B. and Salzberg, S.L. (2012) Fast gapped-read alignment with Bowtie 2. *Nat. Methods*, **9**, 357–9.

2. Li, H., Handsaker, B., Wysoker, A., Fennell, T., Ruan, J., Homer, N., Marth, G., Abecasis, G. and Durbin, R. (2009) The Sequence Alignment/Map format and SAMtools. *Bioinformatics*, **25**, 2078–9.

3. Guo, Y., Mahony, S. and Gifford, D.K. (2012) High resolution genome wide binding event finding and motif discovery reveals transcription factor spatial binding constraints. *PLoS Comput. Biol.*, **8**, e1002638.

4. Ramachandran, P., Palidwor, G. a, Porter, C.J. and Perkins, T.J. (2013) MaSC: mappability-sensitive cross-correlation for estimating mean fragment length of single-end short-read sequencing data. *Bioinformatics*, **29**, 444–50.

5. Rhead, B., Karolchik, D., Kuhn, R.M., Hinrichs, A.S., Zweig, A.S., Fujita, P. a, Diekhans, M., Smith, K.E., Rosenbloom, K.R., Raney, B.J., et al. (2010) The UCSC Genome Browser database: update 2010. *Nucleic Acids Res.*, **38**, D613–9.

6. Thorvaldsdóttir, H., Robinson, J.T. and Mesirov, J.P. (2013) Integrative Genomics Viewer (IGV): high-performance genomics data visualization and exploration. *Brief. Bioinform.*, **14**, 178–92.

7. Gray, K. a, Daugherty, L.C., Gordon, S.M., Seal, R.L., Wright, M.W. and Bruford, E. a (2012) Genenames.org: the HGNC resources in 2013. *Nucleic Acids Res.*, 10.1093/nar/gks1066.

8. Heinz, S., Benner, C., Spann, N., Bertolino, E., Lin, Y.C., Laslo, P., Cheng, J.X., Murre, C., Singh, H. and Glass, C.K. (2010) Simple Combinations of Lineage-Determining Transcription Factors Prime cis-Regulatory Elements Required for Macrophage and B Cell Identities. *Mol. Cell*, **38**, 576–589.

9. Korhonen, J., Martinmäki, P., Pizzi, C., Rastas, P. and Ukkonen, E. (2009) MOODS: fast search for position weight matrix matches in DNA sequences. *Bioinformatics*, **25**, 3181–2.

10. Hunter, J.D. (2007) Matplotlib: A 2D Graphics Environment. *Comput. Sci. Eng.*, **9**, 90–95.

11. Encode, T. and Consortium, P. (2011) A user’s guide to the encyclopedia of DNA elements (ENCODE). *PLoS Biol.*, **9**, e1001046.

12. Du, P. (2009) lumi (Illumina Bead Array Bioconductor Package).

13. Smyth, G.K., Ritchie, M., Thorne, N. and Wettenhall, J. (2008) Linear Models for Microarray Data User ’ s Guide (LIMMA).

14. Care, M.A., Barrans, S., Worrillow, L., Jack, A., Westhead, D.R. and Tooze, R.M. (2013) A microarray platform-independent classification tool for cell of origin class allows comparative analysis of gene expression in diffuse large B-cell lymphoma. *PLoS One*, **8**, e55895.

15. Team, R.D.C. (2008) R: A Language and Environment for Statistical Computing. *Vienna Austria R Found. Stat. Comput.*, **1**, ISBN 3–900051–07–0.

16. Cocco, M., Stephenson, S., Care, M. a, Newton, D., Barnes, N. a, Davison, A., Rawstron, A., Westhead, D.R., Doody, G.M. and Tooze, R.M. (2012) In vitro generation of long-lived human plasma cells. *J. Immunol.*, **189**, 5773–85.

17. Shaffer, A.L., Wright, G., Yang, L., Powell, J., Ngo, V., Lamy, L., Lam, L.T., Davis, R.E. and Staudt, L.M. (2006) A library of gene expression signatures to illuminate normal and pathological lymphoid biology. *Immunol. Rev.*, **210**, 67–85.

18. Subramanian, A., Tamayo, P., Mootha, V.K., Mukherjee, S., Ebert, B.L., Gillette, M.A., Paulovich, A., Pomeroy, S.L., Golub, T.R., Lander, E.S., et al. (2005) Gene set enrichment analysis: a knowledge-based approach for interpreting genome-wide expression profiles. *Proc. Natl. Acad. Sci. U. S. A.*, **102**, 15545–50.

19. Culhane, A.C., Schwarzl, T., Sultana, R., Picard, K.C., Picard, S.C., Lu, T.H., Franklin, K.R., French, S.J., Papenhausen, G., Correll, M., et al. (2010) GeneSigDB--a curated database of gene expression signatures. *Nucleic Acids Res.*, **38**, D716–25.

20. Ngo, V.N., Young, R.M., Schmitz, R., Jhavar, S., Xiao, W., Lim, K.-H., Kohlhammer, H., Xu, W., Yang, Y., Zhao, H., et al. (2011) Oncogenically active MYD88 mutations in human lymphoma. *Nature*, **470**, 115–9.

21. Monti, S., Savage, K.J., Kutok, J.L., Feuerhake, F., Kurtin, P., Mihm, M., Wu, B., Pasqualucci, L., Neuberg, D., Aguiar, R.C.T., et al. (2005) Molecular profiling of diffuse large B-cell lymphoma identifies robust subtypes including one characterized by host inflammatory response. *Blood*, **105**, 1851–61.

22. Hamoudi, R. a, Appert, A., Ye, H., Ruskone-Fourmestraux, A., Streubel, B., Chott, A., Raderer, M., Gong, L., Wlodarska, I., De Wolf-Peeters, C., et al. (2010) Differential expression of NF-kappaB target genes in MALT lymphoma with and without chromosome translocation: insights into molecular mechanism. *Leuk. Off. J. Leuk. Soc. Am. Leuk. Res. Fund, U.K*, **24**, 1487–97.

23. Huang, X., Meng, B., Iqbal, J., Ding, B.B., Perry, A.M., Cao, W., Smith, L.M., Bi, C., Jiang, C., Greiner, T.C., et al. (2013) Activation of the STAT3 signaling pathway is associated with poor survival in diffuse large B-cell lymphoma treated with R-CHOP. *J. Clin. Oncol.*, **31**, 4520–8.

24. Wickham, H. (2011) ggplot2. *Wiley Interdiscip. Rev. Comput. Stat.*, **3**, 180–185.

25. Reich, M., Liefeld, T., Gould, J., Lerner, J., Tamayo, P. and Mesirov, J.P. (2006) GenePattern 2.0. *Nat. Genet.*, **38**, 500–1.

26. Mahony, S. and Benos, P. V (2007) STAMP: a web tool for exploring DNA-binding motif similarities. *Nucleic Acids Res.*, **35**, W253–8.
